# Improved precision of epigenetic clock estimates across tissues and its implication for biological ageing

**DOI:** 10.1186/s13073-019-0667-1

**Published:** 2019-08-23

**Authors:** Qian Zhang, Costanza L. Vallerga, Rosie M. Walker, Tian Lin, Anjali K. Henders, Grant W. Montgomery, Ji He, Dongsheng Fan, Javed Fowdar, Martin Kennedy, Toni Pitcher, John Pearson, Glenda Halliday, John B. Kwok, Ian Hickie, Simon Lewis, Tim Anderson, Peter A. Silburn, George D. Mellick, Sarah E. Harris, Paul Redmond, Alison D. Murray, David J. Porteous, Christopher S. Haley, Kathryn L. Evans, Andrew M. McIntosh, Jian Yang, Jacob Gratten, Riccardo E. Marioni, Naomi R. Wray, Ian J. Deary, Allan F. McRae, Peter M. Visscher

**Affiliations:** 1 Institute for Molecular Bioscience, The University of Queensland, Brisbane, Queensland 4072 Australia; 2 Centre for Genomic and Experimental Medicine, Institute of Genetics and Molecular Medicine, University of Edinburgh, Edinburgh, EH4 2XU UK; 3 Department of Neurology, Peking University, Third Hospital, No. 49, North Garden Road, Haidian District, Beijing, 100191 China; 4 Griffith Institute for Drug Discovery (GRIDD), Griffith University, Brisbane, Australia; 5 Department of Pathology and Biomedical Science, University of Otago, Christchurch, New Zealand; 6 New Zealand Brain Research Institute, Christchurch, New Zealand; 7 Department of Medicine, University of Otago, Christchurch, New Zealand; 8 Brain and Mind Centre, Sydney Medical School, The University of Sydney, Sydney, Australia; 9 Queensland Brain Institute, The University of Queensland, Brisbane, Queensland 4072 Australia; 10 Centre for Cognitive Ageing and Cognitive Epidemiology, University of Edinburgh, Edinburgh, EH8 9JZ UK; 11 Aberdeen Biomedical Imaging Centre, University of Aberdeen, Lilian Sutton Building, Foresterhill, Aberdeen, AB25 2ZD UK; 12 MRC Human Genetics Unit, Institute of Genetics and Molecular Medicine, University of Edinburgh, Edinburgh, EH4 2XU UK; 13 Division of Psychiatry, University of Edinburgh, Royal Edinburgh Hospital, Edinburgh, EH10 5HF UK; 14 Department of Psychology, University of Edinburgh, Edinburgh, EH8 9JZ UK

**Keywords:** DNA methylation, Age prediction, Epigenetic clock, Ageing, Mortality

## Abstract

**Background:**

DNA methylation changes with age. Chronological age predictors built from DNA methylation are termed ‘epigenetic clocks’. The deviation of predicted age from the actual age (‘age acceleration residual’, AAR) has been reported to be associated with death. However, it is currently unclear how a better prediction of chronological age affects such association.

**Methods:**

In this study, we build multiple predictors based on training DNA methylation samples selected from 13,661 samples (13,402 from blood and 259 from saliva). We use the Lothian Birth Cohorts of 1921 (LBC1921) and 1936 (LBC1936) to examine whether the association between AAR (from these predictors) and death is affected by (1) improving prediction accuracy of an age predictor as its training sample size increases (from 335 to 12,710) and (2) additionally correcting for confounders (i.e., cellular compositions). In addition, we investigated the performance of our predictor in non-blood tissues.

**Results:**

We found that in principle, a near-perfect age predictor could be developed when the training sample size is sufficiently large. The association between AAR and mortality attenuates as prediction accuracy increases. AAR from our best predictor (based on Elastic Net, https://github.com/qzhang314/DNAm-based-age-predictor) exhibits no association with mortality in both LBC1921 (hazard ratio = 1.08, 95% CI 0.91–1.27) and LBC1936 (hazard ratio = 1.00, 95% CI 0.79–1.28). Predictors based on small sample size are prone to confounding by cellular compositions relative to those from large sample size. We observed comparable performance of our predictor in non-blood tissues with a multi-tissue-based predictor.

**Conclusions:**

This study indicates that the epigenetic clock can be improved by increasing the training sample size and that its association with mortality attenuates with increased prediction of chronological age.

**Electronic supplementary material:**

The online version of this article (10.1186/s13073-019-0667-1) contains supplementary material, which is available to authorized users.

## Background

Ageing is a major risk for diseases and mortality [1, 2]. Chronological age has been widely used as a marker of ageing due to ease and accuracy of measurement [1]. However, it is not necessarily a good predictor of biological ageing since individuals with the same chronological age can vary in health, especially in later life [3]. Therefore, researchers have attempted to search for biomarkers of ageing that can predict functional capability at a later age [4, 5]. In 2013, Hannum et al. and Horvath built chronological age predictors (termed ‘epigenetic clock’) based on DNA methylation [6, 7]. Subsequently, a number of other DNA methylation-based ‘clocks’ have been developed [8–11]. These clocks utilize age acceleration residuals (AAR, defined as the residuals from regressing predicted age on chronological age) as a biomarker of ageing[7]. Individuals with positive AAR are considered to be biologically older than their chronological age, and vice versa.

A number of studies have identified associations between AAR and mortality, obesity and other health-related traits [12–15]. However, a better way of predicting these health-related traits is developing a predictor with the target trait as a reference [8, 10, 11, 16]. For example, a mitotic clock has been built for tracking the proliferation of cancer [8, 16]. DNAPhenoAge [10] and DNAmGrimAge [11] predictors were developed to predict a composite phenotype (composed of chronological age and clinical markers). Both of these predictors show stronger associations with lifespan and mortality than Horvath’s age predictor [7]. By definition, AAR from a perfect chronological age predictor should be zero. Therefore, no information on inter-individual variation in biological age can be identified based on such a predictor [17]. Nevertheless, whether we can develop a perfect chronological age predictor based on DNA methylation is unknown. Besides, whether the reported associations between AAR and health-related traits (e.g., mortality) are inflated (e.g., by potential confounders) and/or affected by the prediction accuracy of ‘epigenetic clock’ has not been investigated.

In the present study, to investigate whether a perfect DNA methylation-based age predictor is theoretically available, we estimated the proportion of variance of age that could be explained by DNA methylation using a mixed linear model. We developed age predictors based on training sets with various sample sizes using Elastic Net [18] and Best Linear Unbiased Prediction (BLUP) [19]. We calculated AAR based on these age predictors and examined whether its association with mortality is affected by the prediction accuracy and potential confounders. We further investigated the performance of our predictor in samples from tissues other than blood.

## Methods

### Study population

We collected 14 data cohorts with 13,661 samples (13,402 from blood and 259 from saliva) in the age range of 2 to 104 years measured on the DNA methylation HumanMethylation450 chips and Illumina EPIC (850 K) arrays (Table 1). Eight of these cohorts were from the public domain (GEO database) and six datasets from the investigators. The six datasets include Lothian Birth Cohort (LBC) 1921/1936, Brisbane Systems Genomics Study (BSGS), Systems Genomic of Parkinson’s Disease consortium (SGPD), Motor Neuron Disease cohort (MND), and Generation Scotland (GS). Details of samples in BSGS and LBC cohorts can be found in Powell et al. [22] and Deary et al. [20, 21]. GS is a population- and family-based cohort recruited through the National Health Service (NHS) Scotland general practitioner research network [24, 25]. The SGPD cohort is from a collaborative research project on systems genomics of Parkinson’s disease. Similarly, the MND cohort is from a systems genomics study of Motor Neuron Disease in Chinese subjects (see descriptions in Benyamin et al. [23]). DNA methylation beta value at each probe was used for analysis.

**Table 1:** Description of DNA methylation cohorts

Cohort^1^	Sample size^2^	Number of samples with valid age	Mean age (SD)	Age range	Source	Disease
LBC1921 [20, 21]	692	692	82.3 (4.3)	[77.8, 90.6]	Blood	Not available
LBC1936 [20, 21]	2326	2326	72.4 (2.8)	[67.7, 77.7]	Blood	Not available
BSGS [22]	614	614	21.4 (14.1)	[9.9, 74.9]	Blood	Not available
SGPD	1962	1556	67.2 (9.5)	[23.0, 104.0]	Blood	Parkinson’s disease 988, control 974
MND [23]	695	600	45.2 (15.0)	[17.0, 76.0]	Blood	Motor neuron disease (MND) 497, control 198
GS [24, 25]	5101	5100	48.5(14.0)	[18.0, 94.5]	Blood	Not available
GSE72775 [26]	335	335	70.2 (10.3)	[36.5, 90.5]	Blood	Not available
GSE78874 [26]	259	259	68.8(9.7)	[36.0, 88.0]	Saliva	Not available
GSE72773 [26]	310	310	65.6 (13.9)	[35.1, 91.9]	Blood	Not available
GSE72777 [26]	46	46	14.7 (10.4)	[2.2, 35.0]	Blood	Not available
GSE41169 [27]	95	95	31.6 (10.3)	[18.0, 65.0]	Blood	Schizophrenia 62, control 33
GSE40279 [6]	656	656	64.0 (14.7)	[19.0, 101.0]	Blood	Not available
GSE42861 [28]	689	689	51.9 (11.8)	[18.0, 70.0]	Blood	Rheumatoid arthritis 354, control 335
GSE53740 [29]	384	383	67.8(9.6)	[34.0, 93.0]	Blood	Alzheimer’s disease 15, corticobasal degeneration 1, frontotemporal dementia (FTD) 121, FTD/MND 7, progressive supranuclear palsy 43, control 193, unknown 4

^1^*LBC* Lothian Birth Cohort, *BSGS* Brisbane Systems Genomics Study, *SGPD* Systems Genomic of Parkinson’s Disease consortium, *MND* Motor Neuron Disease cohort, *GS* Generation Scotland. Cohorts with prefix GSE are from the GEO database

^2^The number of samples in each cohort. Some samples in LBC were measured from the same individual but at different chronological age

After quality control, we obtained a set of 319,607 probes (called the No Pruned set) for each sample (Additional file 1). The effective number of independent methylation probes was previously reported to be around 200 [30], indicating a dense correlation structure. Therefore, we generated a pruned probe set (128,405 probes) (Additional file 1) and compared its performance in age prediction with that based on No Pruned set. Two cohorts were identified to be outliers in the principal components analysis (PCA) using probes from the No Pruned set (Additional file 1: Figure S1). However, the prediction accuracy in both of these cohorts is not low, and thus, we kept them in the subsequent analysis (Additional file 1).

Most of the training samples of our age predictors are from the blood. To test the performance of our age predictors in non-blood tissues, we downloaded 13 additional cohorts (from GEO database) with samples from tissues other than the blood (Additional file 2: Table S1).

### Estimating the proportion of variance of chronological age explained by DNA methylation

The GS and SGPD samples were used in estimating the proportion of variance of chronological age explained by DNA methylation. Among the 5101 samples in the GS cohort, a subset of 2586 unrelated individuals, with a genetic relationship coefficient below 0.05 and with no shared nuclear family environment, were considered for the analysis. Meanwhile, we selected 1299 unrelated (genetic relationship coefficient < 0.05) individuals with available age information in SGPD. We estimated the proportion of variance of age explained when fitting all probes simultaneously by the restricted maximum likelihood (REML) method implemented in OSCA [31], which is analogous to estimating heritability from SNP data [32].

$$ \boldsymbol{Y}=\boldsymbol{Xu}+\boldsymbol{e} $$

where ***Y*** is an *n × 1* vector of phenotype values (here chronological age) with *n* being the sample size. ***X*** is an *n × m* matrix of standardized DNA methylation measures of all *m* probes, ***u*** is an *m × 1* vector of the joint effects of all probes on the phenotype, and ***e*** is an *n × 1* vector of residuals. Both ***u*** and ***e*** are random effects with $\boldsymbol{u}\sim \boldsymbol{N}\left(\mathbf{0},\boldsymbol{I}{\sigma}_u^2\right)$ and $\boldsymbol{e}\sim \boldsymbol{N}\left(\mathbf{0},\boldsymbol{I}{\sigma}_e^2\right)$*,* ${\sigma}_u^2$ and ${\sigma}_e^2$ can be estimated by REML. The proportion of variance of chronological age explained by all DNA methylation probes is defined as:

$$ {\rho}^2=\frac{\sigma_u^2}{\sigma_u^2+{\sigma}_e^2} $$

*ρ*^2^ = 0 means that DNA methylation is not associated with phenotypic differences between individuals; *ρ*^2^ = 1 means all the variation in the phenotype can be explained by the joint effects of DNA methylation from all probes.

### Building age predictors

We generated 65 training sets from the 14 cohorts. Each training set has a certain number (ranging between 1 and 13) of cohorts sampled from the 14 cohorts, and the unselected cohorts were used as test set(s). For each number, we repeated random sampling five times (Additional file 2: Figure S1). For example, there will be five training sets composed of ten cohorts, and the ten cohorts in each training set were sampled from the 14 cohorts randomly. In total, 65 (13 × 5) training sets were generated.

Based on each training set, we built our predictors using two methods, namely Elastic Net and BLUP. Both of them are based on a linear regression:

$$ \boldsymbol{Y}=\boldsymbol{X}\boldsymbol{\beta } +\boldsymbol{e} $$

where ***Y*** is an *n × 1* vector of chronological age with *n* being the sample size. ***X*** is an *n × m* matrix of DNA methylation measures of all *m* probes, whereby *X*_*ij*_ is the DNA methylation of individual *i* at probe *j*, and ***e*** is the Gaussian error. The two methods differ in how they select probes that are associated with age and how their effects are estimated (Additional file 1). BLUP would perform better than Elastic Net when there are many predictors (probes), all with non-zero effects on the target trait and effects drawn from a normal distribution. However, this method needs a large sample size to estimate small effect sizes. It is not always the case that there are many predictors associated with a trait.

We implemented two estimates to evaluate the performance of our age predictors: (1) correlation between predicted age and chronological age in the test data set and (2) root mean square error (RMSE) of the predicted age in the test data set. Correlation indicates the strength of a linear relationship between the predicted age and chronological age, and RMSE reveals the variation of the difference between predicted and chronological age.

The relationship between chronological age and DNA methylation could be nonlinear [33]. We selected eight DNA methylation cohorts with a sample size larger than 600 to evaluate the impact of data transformation in age prediction: LBC1921, LBC1936, GS, BSGS, SGPD, MND, GSE40279, and GSE42861. For each cohort, we randomly selected 70% of the samples as a training set and the remaining 30% were used as the test set. Only 50,000 randomly selected probes were used for computational efficiency. Power parameter *λ* (ranges from 0.1 to 2 with 0.05 as the interval) was used to transform the original beta value of DNA methylation BV to BV^*λ*^. Only BLUP was used for age prediction because of its low bias. DNA methylation *M* value, arcsine square root transformed methylation beta value, and log transformed methylation beta value were also used to compare to raw DNA methylation beta value in prediction accuracy.

### Association between age acceleration residual and mortality

We detected the association between age acceleration residual (AAR) and mortality by using the Cox proportional hazards regression model with age at sample collection, sex plate, array, position on the array and hybridization date as the covariates (all treated as fixed effect factors). Samples from the updated data in Marioni et al. [12]: LBC1921 (wave one, *N* = 436, *N*_deaths_ = 386) and LBC1936 (wave one, *N* = 906, *N*_deaths_ = 214) were used in this analysis. AAR was calculated based on age predictors excluding LBC1921/LBC1936 as part of the training set (sample size ranges from 335 to 12,710). Cox models were performed utilizing the ‘survival’ library in R [34]. We applied a sensitivity analysis by additionally including the measured cell count of each white blood cell type (basophils, eosinophils, monocytes, lymphocytes, and neutrophils) as covariates in the Cox model. The change of test statistics of AAR before and after fitting these covariates was quantified.

Variation in cellular compositions is known to be associated with DNA methylation [35]. We examined whether AAR-associated CpG sites were enriched in the probes that show heterogeneity in DNA methylation across cell types (72,393 cellular heterogeneity probes) [36] using the Fisher exact test. We calculated AAR for samples from LBC1936 wave one using predictors without LBC1936 in the training set. Based on AAR from each predictor, we estimated its association with DNA methylation at each CpG site. AAR-associated CpG sites were defined as the probes with *P* value smaller than Bonferroni-corrected *P* value threshold (*P* = 0.05/319,607).

## Results

### Estimation of variation in age from using all probes

The proportion of variance of age explained by DNA methylation was close to 1 in both cohorts based on REML analysis (GS: proportion explained = 1, SE = 0.0036; SGPD: proportion explained = 0.99, SE = 0.058) (‘Methods’ section), suggesting that variation of chronological age between individuals could be entirely explained by the joint effect of DNA methylation from all CpG sites. It indicates that a perfect age predictor can in principle be developed based on DNA methylation data if all probe associations are estimated without error. To demonstrate that this result is not caused by overestimation, we undertook a permutation test using the same cohorts. We shuffled the ages across individuals and found that DNA methylation did not explain any significant amount of variation in GS (proportion explained = 0, SE = 0.0030) and SGPD (proportion explained = 0.0079, SE = 0.013).

### Age predictors with different prediction accuracy

**Fig. 1:**
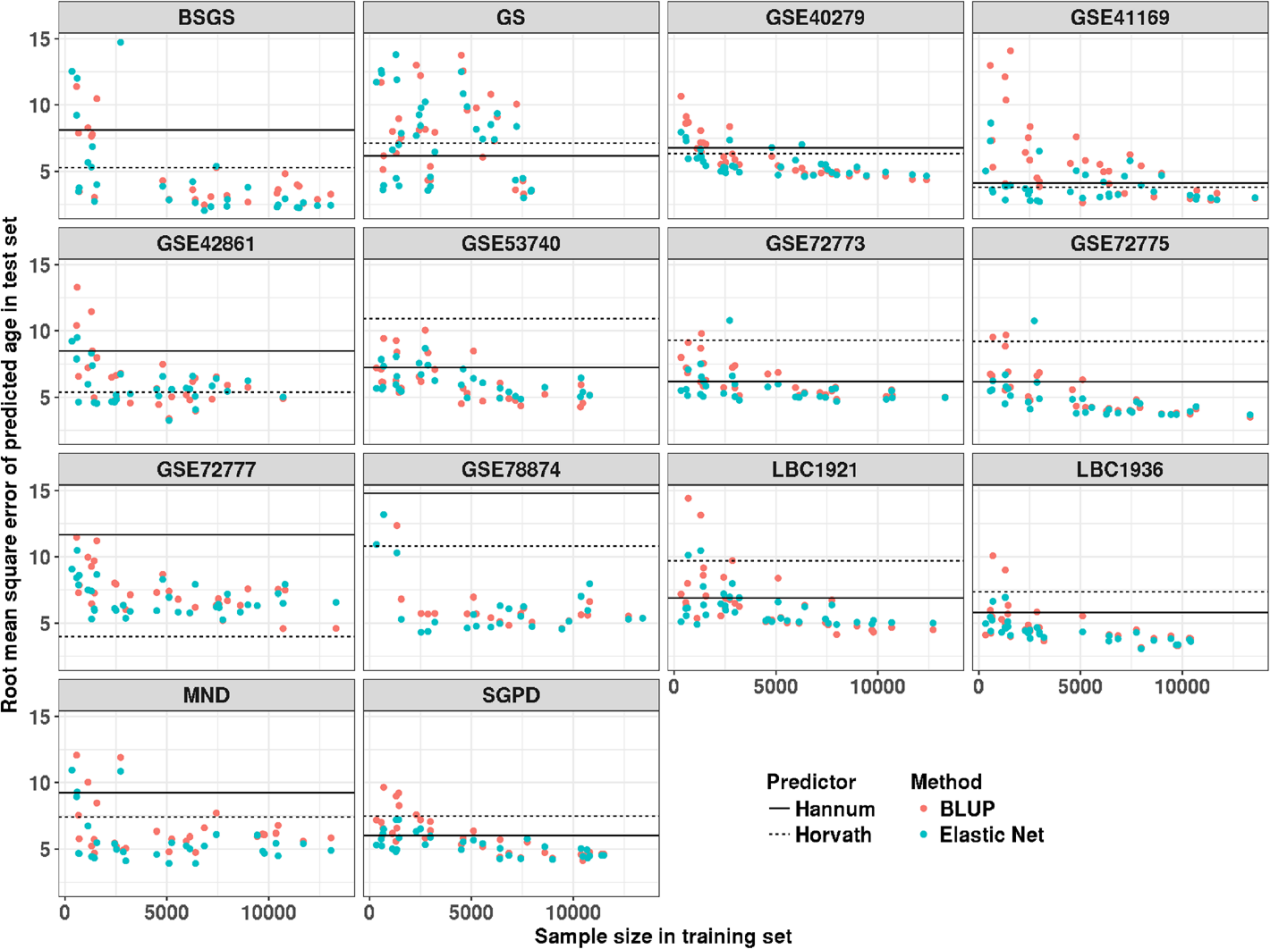
The relationship between training sample size and predictor error measured at the square root of the mean squared error (RMSE) in test data sets. Each point represents the RMSE of the test result based on predictors with different sample size and methods. Points with RMSE larger than 15 are excluded. Prediction results from Horvath are marked as black dash line, and the black solid line represents prediction result from Hannum’s age predictor

Based on each training set (65 in total), we built our predictors using BLUP and Elastic Net (‘Methods’ section). Results on the test sets show that both methods have a decrease of RMSE (Fig. 1) and an increase of correlation (Additional file 2: Figure S2) when the training sample size increased. The smallest RMSE based on Elastic Net was 2.04 years, which is lower than that based on Hannum’s and Horvath’s age predictors (Additional file 2: Figure S3). This method gave better results with RMSE relative to BLUP for small training sample size, although the difference with BLUP became smaller with increased sample size (Additional file 2: Figure S4). The imperfect prediction performance (RMSE = 2.04) of the predictor in this study could be caused by an insufficient number of training samples and/or different batch effects between the training and test dataset. Analogous to estimation and prediction of complex traits using SNPs, prediction accuracy is expected to be less than the total variance explained by all features. They are the same when effect sizes are estimated without error.

Apart from the sample size, we found increasing the age range of training set and the age similarity between training and test set can improve the prediction accuracy (Additional file 2: Table S2 & S3). No steady improvement could be achieved by using transformed beta value (‘Methods’ section, Additional file 2: Figure S5 & S6). In addition, higher RMSE and lower correlation can always be observed for prediction results based pruned set than full probe set (Additional file 2: Figure S7). The overlap (and correlation) is small between 514 probes in our predictor (selected by Elastic Net, based on 13,566 training samples) and that in the age predictors of Hannum (30 in common) and Horvath (11 in common) (Additional file 2: Figure S8). Probes in these two predictors were found to be redundant for age prediction (Additional file 2: Figure S9), and better prediction accuracy can still be observed after removing these probes (Additional file 2: Figure S10).

### Association between AAR and mortality

**Fig. 2:**
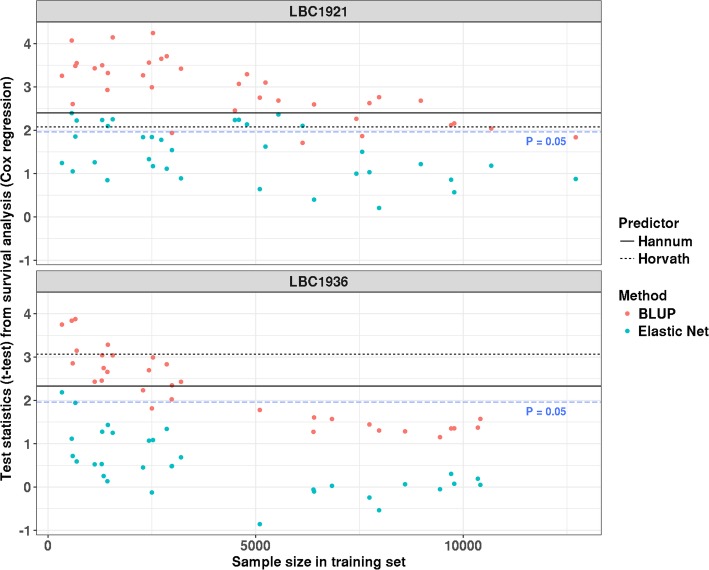
Relationship between the training sample size and the test statistics (*t* test) from the association between age acceleration residual (AAR) and mortality. Each point represents the test statistic from the survival analysis based on the predicted ages from predictors with different training sample sizes

Based on samples from wave one of both LBC1921 and LBC1936, we observed a decrease of the test statistics (*z*-statistic) for the association between AAR and death (from the Cox regression) with increasing sample size in training data set (Fig. 2). For AAR calculated from the predictor with the largest training sample size, it was not associated with the mortality in either LBC1921 or LBC1936 using BLUP (LBC1921: hazard ratio = 1.20, 95% CI 0.99–1.46, *P* value = 0.066; LBC1936: hazard ratio = 1.25, 95% CI 0.95–1.64, *P* = 0.12) or Elastic Net (LBC1921: hazard ratio = 1.08, 95% CI 0.91–1.27, *P* = 0.38; LBC1936: hazard ratio = 1.00, 95% CI 0.79–1.28, *P* = 0.96) (Table 2). In contrast, results based on the age predictors of Hannum and Horvath were significant (*P* < 0.05, Table 2).

**Table 2:** Summary details of two LBC cohorts and the relationship between all-cause mortality and predicted age from different methods (before and after cell count correction)

	LBC1921 wave one	LBC1936 wave one
*N*	436	906
*N*_deaths_	386	214
Chronological age, mean (SD)^1^	79.1 (0.6)	69.5 (0.8)
Before cell count correction		
Hannum, mean (SD)	80.3 (6.2)	71.3 (5.7)
Hannum, hazard ratio (*P* value, 95% CI)^2^	1.12 (0.016, 1.02–1.23)	1.18 (0.020, 1.02–1.37)
Horvath, mean (SD)	73.8 (6.9)	66.1 (6.4)
Horvath, hazard ratio (*P* value, 95% CI)	1.09 (0.038, 1.00–1.20)	1.19 (0.0022, 1.06–1.32)
Elastic Net, mean (SD)^3^	77.4 (3.6)	72.5 (3.2)
Elastic Net, hazard ratio (*P* value, 95% CI)	1.08 (0.38, 0.91–1.27)	1.00 (0.96, 0.79–1.28)
BLUP, mean (SD)^3^	77.3 (3.3)	72.5 (2.8)
BLUP, hazard ratio (*P* value, 95% CI)	1.20 (0.066, 0.99–1.46)	1.25 (0.12, 0.95–1.64)
After cell count correction		
Hannum, hazard ratio (*P* value, 95% CI)	1.10 (0.057, 1.00–1.21)	1.11 (0.15, 0.96–1.29)
Horvath, hazard ratio (*P* value, 95% CI)	1.07 (0.13, 0.98–1.17)	1.14 (0.032, 1.01–1.28)
Elastic Net, hazard ratio (*P* value, 95% CI)^3^	1.07 (0.39, 0.91–1.27)	1.03 (0.79, 0.82–1.31)
BLUP, hazard ratio (*P* value, 95% CI)^3^	1.21 (0.05, 1.00–1.48)	1.21 (0.17, 0.92–1.60)

^1^Mean (predicted) age and its standard deviation

^2^Hazard ratio, *P* value, and 95% confidence interval from the survival analysis based on the predicted age. Hazard ratios were expressed per 5 years of methylation age acceleration

^3^Both results of Elastic Net and BLUP were based on the age predictor with the largest training sample size (sample size = 10,411 for LBC1936 and sample size = 12,710 for LBC1921)

**Fig. 3:**
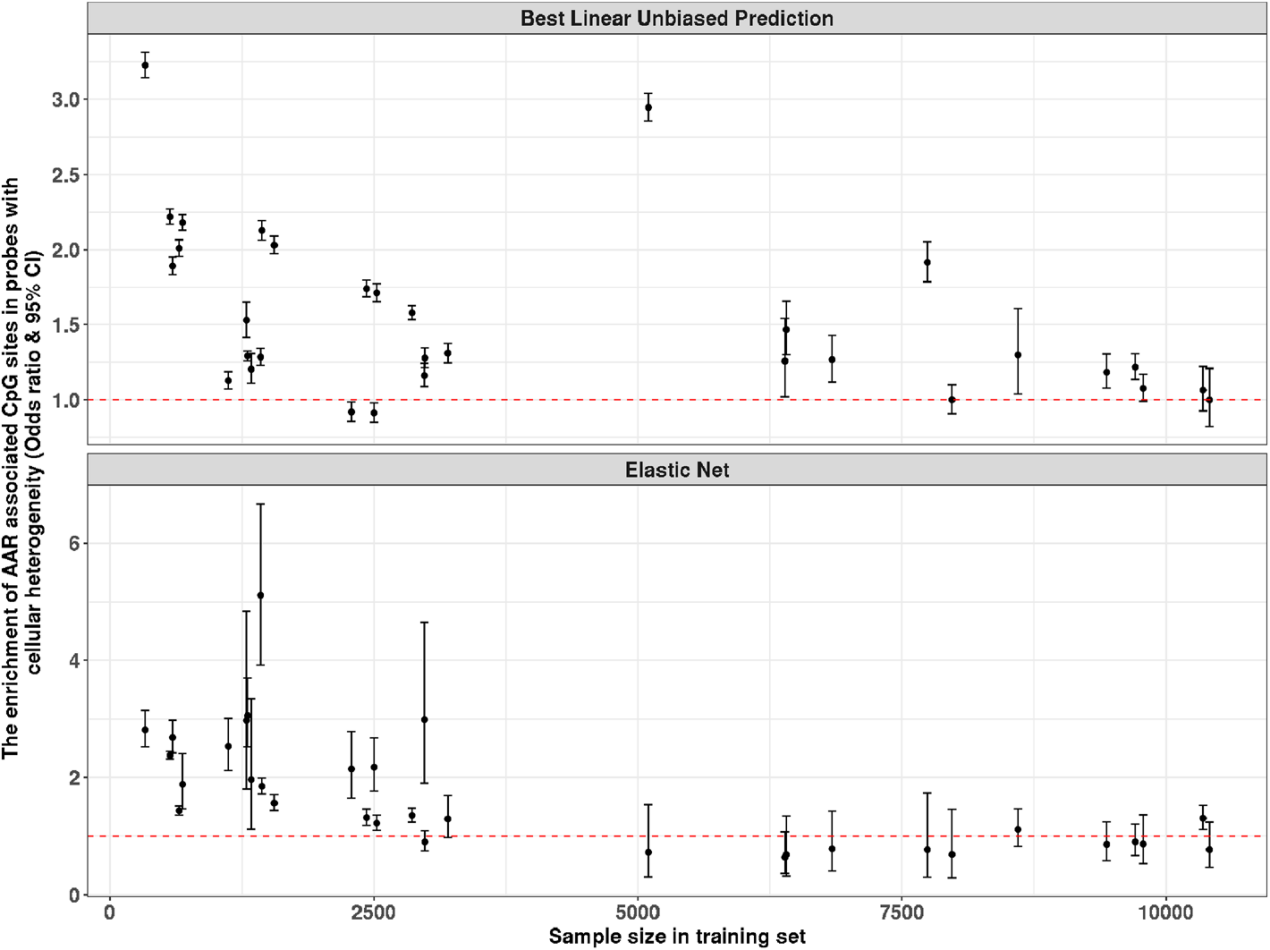
The change of odds ratio from the enrichment test with the increase of training sample size (excluding LBC1936). The enrichment test examines whether AAR-associated CpG sites are enriched in probes with cellular heterogeneity

AAR-associated CpG sites from age predictors of Hannum (odds ratio = 3.85, 95% CI 3.71–4.00, *P* < 2.2 × 10^−16^) and Horvath (odds ratio = 2.53, 95% CI 2.45–2.61, *P* < 2.2 × 10^−16^) were found to be enriched in probes that show heterogeneity in DNA methylation across cell types (‘Methods’ section), suggesting AAR from these two predictors is affected by the cellular compositions. A decrease of the odds ratio from the enrichment test was observed with the increase of training sample size for both Elastic Net and BLUP-based age predictors (Fig. 3). No significant enrichment (Elastic Net: odds ratio = 0.78, 95% CI 0.47–1.23, *P* = 0.33; BLUP: odds ratio = 1, 95% CI 0.82–1.21, *P* = 1.00) was found for the age predictors based on the largest training sample size (Table 3).

**Table 3:** Enrichment test on the AAR-associated CpG sites from different methods based upon samples from LBC1936 wave one

	Number of significant associations (*P* < 0.05/319,607)	*λ*_median_	Number of CpG sites showing significant cellular heterogeneity	Odds ratio (*P* value, 95% CI)^1^
Hannum	12,015	3.6	4958	3.85 (*P* < 2.2 × 10^−16^, 3.71–4.00)
Horvath	18,847	5.4	5955	2.53 (*P* < 2.2 × 10^−16^, 2.45–2.61)
Elastic Net^2^	159	2.1	21	0.78 (*P* = 0.33, 0.47–1.23)
BLUP^2^	793	2.6	130	1.00 (*P* = 1.0, 0.82–1.21)

^1^The odds ratio for the enrichment of EWAS significant CpG sites in the probe set showing significant cellular heterogeneity

^2^Both results of Elastic Net and BLUP were based on the age predictor with the largest training sample size (training set without LBC1936, sample size = 10,411)

Apart from AAR, cellular compositions are also related to mortality [37], which suggests it could be a confounder in the association between AAR and mortality. We re-ran the analysis based on AAR adjusting for measured white blood cell (WBC) counts (basophils, eosinophils, monocytes, lymphocytes, and neutrophils) (‘Methods’ section). A decrease of the test statistics (from the Cox regression) after correcting for the WBC counts was observed, especially when the training sample size is small (Additional file 2: Figure S11). After adjustment for WBC, none of the associations remained significant (*P* < 0.05) except for the association in LBC1936 based on the predictor of Horvath (*P* = 0.032). Nevertheless, the significance of this association did not pass the Bonferroni-corrected *P* value threshold (*P* = 0.05/4) (Table 2).

### Age prediction in non-blood tissues

**Fig. 4:**
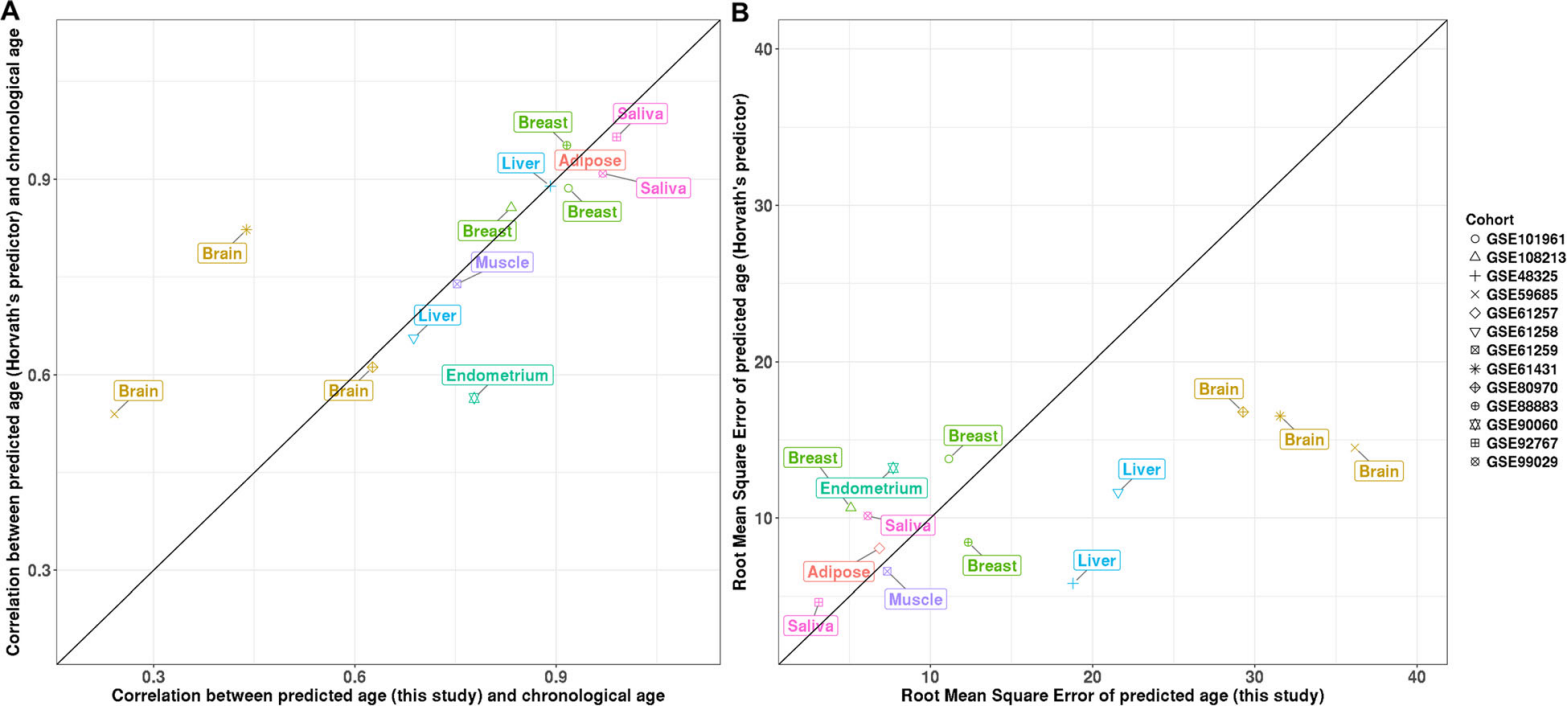
Comparison of prediction performance (**a** correlation and **b** root mean square error) between the predictor from this study (based on Elastic Net) and Horvath’s age predictor in non-blood samples

The majority of our samples are from the blood, and we observed a significant improvement in the prediction results for the samples from saliva when more blood samples were included in the training set (Fig. 1, Additional file 2: Figure S5). This increase is expected since samples from saliva were reported to exhibit more than 80% contamination by immune cells [38]. We also quantified the performance of our predictor in other non-blood tissues based on samples from 13 additional data sets (Additional file 2: Table S1). We compared the performance of our predictor (based on Elastic Net) with Horvath’s age predictor (based on Elastic Net) in these cohorts. Horvath’s age predictor is a pan-tissue epigenetic clock (training samples were from 51 tissues and cell types). It has a good tolerance for tissue specificity since DNA methylation on the selected CpG sites by his predictor was related to age across the tissues and cell types in his training dataset. We found that our predictor has better performance in samples from the endometrium and saliva, in terms of the Pearson correlation between predicted age and chronological age (Fig. 4a). On the other hand, Horvath’s age predictor outperformed our predictor in samples from the brain. Their performance in other tissues (breast, liver, adipose, and muscle) was similar, even though training samples in our predictor are not from these tissues. A similar pattern was observed when comparing the RMSE between these two predictors (Fig. 4b).

## Discussion

We investigated the relationship between the prediction accuracy of a DNA methylation-based age predictor (‘epigenetic clock’) and its application as a biomarker of ageing. Age predictors with various prediction performance were built based on datasets with different sample sizes (ranging from *N* = 335 to 13,566). We used Cox regression to detect the association between age acceleration residuals (AAR, from different age predictors) and mortality based on samples from LBC1921 and LBC1936. We observed a decrease in the significance of the association between AAR and mortality with the improvement of the age predictor. No significant (*P* < 0.05) associations were found based on the age predictor with the largest training sample size (Table 2), suggesting the improved prediction of chronological age from DNA methylation limits it as a biomarker of ageing. Our age predictor from the largest training set of 13,566 individuals is available online [39] (see Declarations).

Among the 13,661 samples from 14 cohorts in this study, 2088 were known to have a disease. DNA methylation at a few CpG sites might be different between these samples and others. Such probes would not be selected into a chronological age predictor when they are not related to age. Even if they are age-related, these probes would be weighted less (or still unselected) since their associations with the disease could affect their prediction accuracy on chronological age. Assigning these probes small effect sizes (or removing them) would decrease the prediction error in the training process. Our results from randomization training and test sets show that our age predictors do not appear to be biased by the disease status.

Notwithstanding the highly correlated pattern of DNA methylation across the genome, we observed a decline of prediction accuracy when using a correlation pruned probe set, so that including more probes in the training model is beneficial, especially when the training sample size is small (Additional file 2: Figure S7). The improvement of prediction accuracy could be explained by the decrease of noise effect (such as batch effects) of DNA methylation in age prediction since using more probes can reduce the unexpected impact of the noise. It could also be caused by the existence of many probes with a small correlation with age, and the cumulative effect of these may be lost when using a pruned set of probes.

The AAR-associated probes from the age predictors of Horvath and Hannum were enriched in CpG sites showing DNA methylation heterogeneity across cell types, suggesting AAR from these predictors is affected by the variation in cellular composition. The sensitivity analysis confirmed that no significant (*P* < 0.05/4) associations were observed after adjusting for white blood cell counts (Table 2). This demonstrates that the difference in the cellular makeup of the samples in our test sets is a confounder in the association between AAR from the Hannum/Horvath age predictors and mortality. This result was not consistent with what has been reported by the previous study. Chen et al. demonstrated that AAR still predicts mortality after adjusting for blood cell counts in a large meta-analysis. [15] However, it should be noted that their blood cell counts were estimated based on DNA methylation, but not measured by the experiment. Hence, the actual cellular composition could still affect the association between AAR and mortality. It is also worth noting that the insignificant result in this study could be caused by lack of power. The association between AAR and mortality was merely examined in two cohorts (LBC1921 and LBC1936). More datasets with measured white blood cell counts are needed to increase detection power.

Our results show that improving the prediction accuracy of an age predictor would reduce the effect of confounders and thereby attenuate the association between AAR and death (Fig. 2). This decrease could be caused by the loss of biological age-associated CpGs in an improved epigenetic clock. It should be noted that building a biological age predictor is difficult since there is no clear definition of biological age. Nevertheless, one of the essential features of biological age is its ability to indicate the different ageing rates between individuals with the same chronological age. A previous study has reported a number of CpG sites that show variation in the longitudinal changing rates between individuals [40]. Utilizing these probes to build a biological age clock might be useful. An alternative approach is developing a predictor for biological age-related traits (e.g., life expectancy [41]), but not for biological age itself.

Although most of the samples in our age predictor are from the blood, it showed good out-of-sample prediction performance in samples from non-blood tissues. Compared with Horvath’s age predictor, we observed larger correlations (between predicted age and chronological age) and smaller RMSE in samples from the saliva and endometrium, but lower correlations and larger RMSE in samples from the brain. These smaller correlations (and larger RMSE) are expected since a large proportion (23.4%) of training samples in Horvath’s age predictor are from the brain. Moreover, these two predictors have similar performance in other tissues. The CpG sites in our age predictor were selected based on their associations with chronological age in blood samples. And Horvath’s age predictor used CpG sites with DNA methylation associated with chronological age across tissues and cell types. The comparable performance of these two predictors implies that most of the age-associated DNA methylation sites in the blood also change along with age in non-blood tissues.

## Conclusions

Our results have several implications for the utility of DNA methylation patterns of age as biomarkers of ageing. From the REML analysis on the SGPD and GS cohorts, we estimated that almost 100% of the variation in chronological age in those samples could be effectively captured by all the DNA methylation probes on the arrays. This implies that a near-perfect predictor of chronological age can be built based on a very large training set. Our results showing that larger sample sizes lead to a more accurate prediction is consistent with this implication. The association between AAR and mortality is confounded by the variation in cellular composition (i.e., white blood cell counts), especially when AAR is from of an age predictor (‘epigenetic clock’) with low performance. Overall, these results suggest that caution is warranted when interpreting estimates from these epigenetic clocks as an indicator of mortality or lifespan.

## Additional files

Additional file 1: Quality Control steps for DNA methylation and details of the prediction methods used in this study. **Figure S1.** Principal component (PC) 1 v.s. PC 2. Two PCs are from the PCA analysis on the samples from 14 selected cohorts. (DOCX 342 kb)

Additional file 2: **Figure S1**. The generation of a training set based on selected cohorts. **Figure S2**. The correlation between the predicted age and chronological age in the test data set. **Figure S3**. The comparison between chronological age and predicted age based on different studies. **Figure S4.** The difference between BLUP and Elastic Net with the increase of sample size in training data set. **Figure S5.** Improvement of age prediction based on power transformed DNA methylation. **Figure S6.** Comparison of age prediction based on arcsine square root transformed DNA methylation, log transformation, DNA methylation M value, and DNA methylation beta value. **Figure S7.** The comparison between the full probe and pruned probe sets. **Figure S8.** The correlations of DNA methylation between probes selected by Elastic Net (based on 13,566 training samples) in this study and those in Horvath’s and Hannum’s age predictors. **Figure S9.** The comparison of prediction accuracy before and after removing probes from probes in the Hannum and Horvath predictors. **Figure S10.** The prediction accuracy of predictors without DNA methylation probes in Hannum’s and Horvath’s Age predictors. **Figure S11.** Relationship between the training sample size and the change of test statistics before and after correcting for the cell counts. **Table S1.** Description of 13 DNA methylation cohorts with non-blood samples. **Table S2.** The contributions of three factors (training sample size, the absolute mean age difference between training data set and test data set, and standard deviation of age from training data set) on the prediction accuracy (RMSE). **Table S3.** The contributions of three factors (training sample size, the absolute mean age difference between training data set and test data set, and standard deviation of age from training data set) on the prediction accuracy (correlation). (DOCX 2106 kb)

## Data Availability

Our age predictor from the largest training set of 13,566 individuals is available online: https://github.com/qzhang314/DNAm-based-age-predictor [39]. GSE40279 [6], GSE72775 [26], GSE78874 [26], GSE72773 [26], GSE72777 [26], GSE41169 [27], GSE42861 [28] and GSE53740 [29] are from GEO database.

## Abbreviations

AAR: Age acceleration residual
LBC: Lothian Birth Cohort
BLUP: Best linear unbiased prediction
GEO: Gene Expression Omnibus
BSGS: Brisbane Systems Genomics Study
SGPD: Systems Genomic of Parkinson’s Disease consortium
MND: Motor Neuron Disease cohort
GS: Generation Scotland
PCA: Principal components analysis
REML: Restricted maximum likelihood
RMSE: Root mean square error
WBC: White blood cell
