# Supplementary material for: Improved precision of epigenetic clock estimates across tissues and its implication for biological ageing
Source: Genome Med. 2019 Aug 23;11:54. doi: 10.1186/s13073-019-0667-1 (PMC6708158; doi:10.1186/s13073-019-0667-1)

**Quality Control**

Probes with call rate less than 0.95 were removed, and probes found to contain SNPs or potentially cross-hybridizing to different locations were excluded from further analysis.[1] A set of 319,607 probes remained in total (called No Pruned set). Pruning was performed by removing one of two probes on the same chromosome when their correlation (R^2^) was higher than an arbitrarily selected threshold of 0.2; this resulted in a set of 128,405 probes (called Pruned set). Both sets were used for further analysis. DNA methylation Beta value was standardized by removing the mean value and divided by the standard deviation for each sample.

Since DNA methylation is sensitive to batch effects, cell type and tissue type[2], we applied a principal components analysis (PCA, using probes from the No Pruned Set) on the samples from these 14 cohorts to assess the presence of any “outlier” cohorts. All the cohorts were closely matched with the exception of GSE78874 and GS (**Figure S1)**. Samples in GSE78874 were from saliva instead of blood, and the samples in GS were measured using Illumina EPIC arrays instead of 450K DNA methylation arrays. To investigate if this difference could potentially adversely influence performance in age prediction for these two cohorts, we used a “leave-one-cohort-out” strategy to leave these two cohorts out as the test set separately and built the age predictor based on the remaining cohorts. We found both of them to have good prediction accuracy (GS: R = 0.98, RMSE = 3.52, GSE78874: R = 0.88, RMSE = 5.39), indicating a small difference between these two cohorts and other cohorts in age prediction. We used all cohorts for subsequent analyses.

**Prediction methods**

For each training set, we built our predictors based on two methods, namely Elastic Net and best linear unbiased predictor (BLUP). Elastic Net was previously used by Horvath[3] and Hannum *et al.[4]* to build their age predictors and BLUP was used to predict age in Peters *et al.[5]*

Elastic Net is a regularized regression method, it seeks to find the $\beta$ that minimize the objective function defined as:

$L\left( \alpha,\beta\right)={||Y-X\beta||}^{2}+\lambda(\frac{1-\alpha}{2}\left| \left| \beta\right| \right|_{2}^{2}+\alpha\left| \left| \beta\right| \right|_{1})$

$\left| \left| \beta\right| \right|_{1}$ is defined as $\sum_{j=1}^{n} |\beta_{j}|$ and $\left| \left| \beta\right| \right|_{2}^{2}$ equals $\sum_{j=1}^{n} {\beta_{j}}^{2}$, with *n* the number of probes. $\alpha$ and lambda are regularisation parameters, $\alpha$ is set to 0.5 and $\lambda$ is chosen based on cross-validation. We used the implementation of Elastic Net from the Python package glmnet [6].

BLUP is a special case of ridge regression with a fixed $\lambda$, $\beta$ can be calculated directly based on the following equation:

$\hat{\beta}={(X^{'}X+\lambda I)}^{-1}X^{'}Y$ with $\lambda=\frac{\sigma_{e}^{2}}{\sigma_{u}^{2}}$,

$\sigma_{u}^{2}$ the variance of the effect size of the probe set, and $\sigma_{e}^{2}$ the variance of the residuals. We used the R package rrBLUP[7] to build the age predictor, and $\sigma_{u}^{2}$ and $\sigma_{e}^{2}$ were estimated using the REML analysis implemented in this package.

**Reference**

1. Price EM, Cotton AM, Lam LL, Farre P, Emberly E, Brown CJ, Robinson WP, Kobor MS: **Additional annotation enhances potential for biologically-relevant analysis of the Illumina Infinium HumanMethylation450 BeadChip array.** *Epigenetics & Chromatin* 2013, **6**.

2. Houseman EA, Accomando WP, Koestler DC, Christensen BC, Marsit CJ, Nelson HH, Wiencke JK, Kelsey KT: **DNA methylation arrays as surrogate measures of cell mixture distribution.** *Bmc Bioinformatics* 2012, **13**.

3. Horvath S: **DNA methylation age of human tissues and cell types.** *Genome biology* 2013, **14:**3156.

4. Hannum G, Guinney J, Zhao L, Zhang L, Hughes G, Sadda S, Klotzle B, Bibikova M, Fan J-B, Gao Y: **Genome-wide methylation profiles reveal quantitative views of human aging rates.** *Molecular cell* 2013, **49:**359-367.

5. Peters MJ, Joehanes R, Pilling LC, Schurmann C, Conneely KN, Powell J, Reinmaa E, Sutphin GL, Zhernakova A, Schramm K: **The transcriptional landscape of age in human peripheral blood.** *Nature communications* 2015, **6**.

6. **Glmnet for Python** [<http://www.stanford.edu/~hastie/glmnet_python/>]

7. Endelman JB: **Ridge regression and other kernels for genomic selection with R package rrBLUP.** *The Plant Genome* 2011, **4:**250-255.

# Figure S1: Principal component (PC) 1 v.s. PC 2. Two PCs are from the PCA analysis on the samples from 14 selected cohorts.


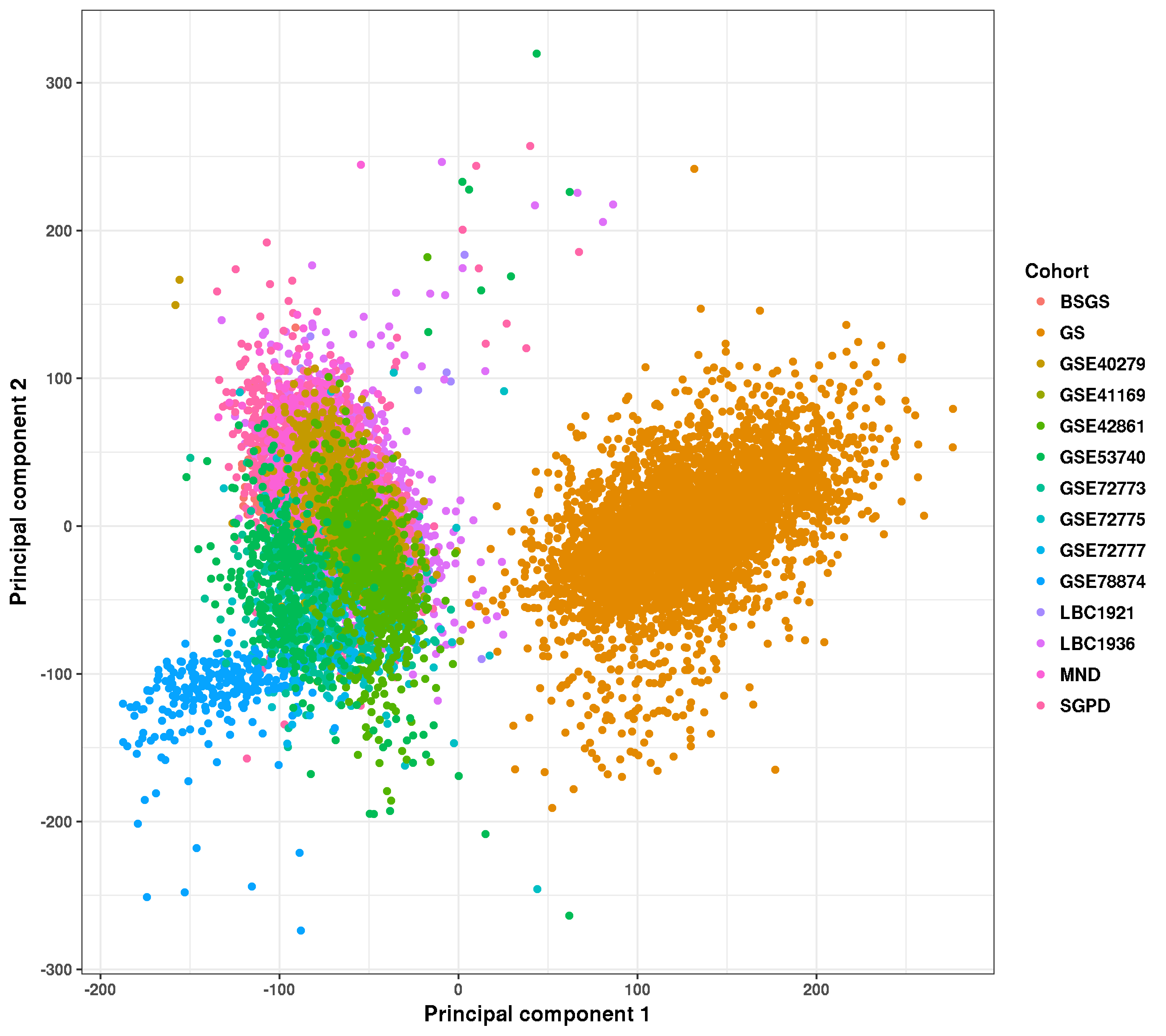

Supplement: Supplementary file 1 — Quality Control steps for DNA methylation and details of the prediction methods used in this study. Figure S1. Principal component (PC) 1 v.s. PC 2. Two PCs are from the PCA analysis on the samples from 14 selected cohorts. (DOCX 342 kb) [file 13073_2019_667_MOESM1_ESM.docx]
