# Supplementary material for: Improved precision of epigenetic clock estimates across tissues and its implication for biological ageing
Source: Genome Med. 2019 Aug 23;11:54. doi: 10.1186/s13073-019-0667-1 (PMC6708158; doi:10.1186/s13073-019-0667-1)
Supplement: Supplementary file 2 — Figure S1. The generation of a training set based on selected cohorts. Figure S2. The correlation between the predicted age and chronological age in the test data set. Figure S3. The comparison between chronological age and predicted age based on different studies. Figure S4. The difference between BLUP and Elastic Net with the increase of sample size in training data set. Figure S5. Improvement of age prediction based on power transformed DNA methylation. Figure S6. Comparison of age prediction based on arcsine square root transformed DNA methylation, log transformation, DNA methylation M value, and DNA methylation beta value. Figure S7. The comparison between the full probe and pruned probe sets. Figure S8. The correlations of DNA methylation between probes selected by Elastic Net (based on 13,566 training samples) in this study and those in Horvath’s and Hannum’s age predictors. Figure S9. The comparison of prediction accuracy before and after removing probes from probes in the Hannum and Horvath predictors. Figure S10. The prediction accuracy of predictors without DNA methylation probes in Hannum’s and Horvath’s Age predictors. Figure S11. Relationship between the training sample size and the change of test statistics before and after correcting for the cell counts. Table S1. Description of 13 DNA methylation cohorts with non-blood samples. Table S2. The contributions of three factors (training sample size, the absolute mean age difference between training data set and test data set, and standard deviation of age from training data set) on the prediction accuracy (RMSE). Table S3. The contributions of three factors (training sample size, the absolute mean age difference between training data set and test data set, and standard deviation of age from training data set) on the prediction accuracy (correlation). (DOCX 2106 kb) [file 13073_2019_667_MOESM2_ESM.docx]

# Figure S1: The generation of a training set based on selected cohorts. An age predictor was built based on this set using two methods. The unselected cohorts were used as test sets, separately. Each square represents one cohort.


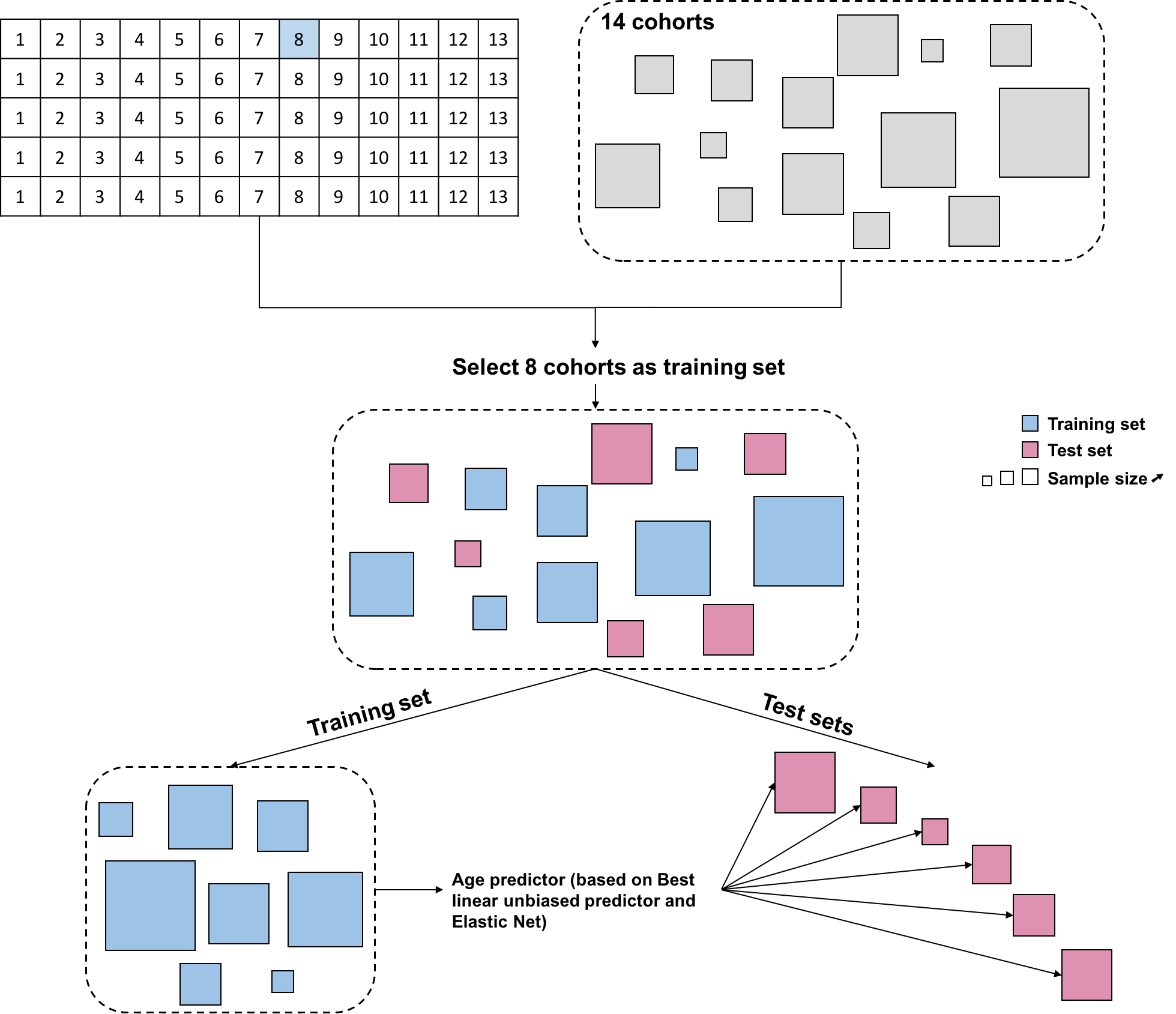


# Figure S2: The correlation between the predicted age and chronological age in the test data set. Each point represents the correlation between predicted age and chronological age based on predictors with different training sample size and methods. Prediction results from Horvath are marked as black dash line, and black solid line represents prediction result from Hannum’s age predictor.


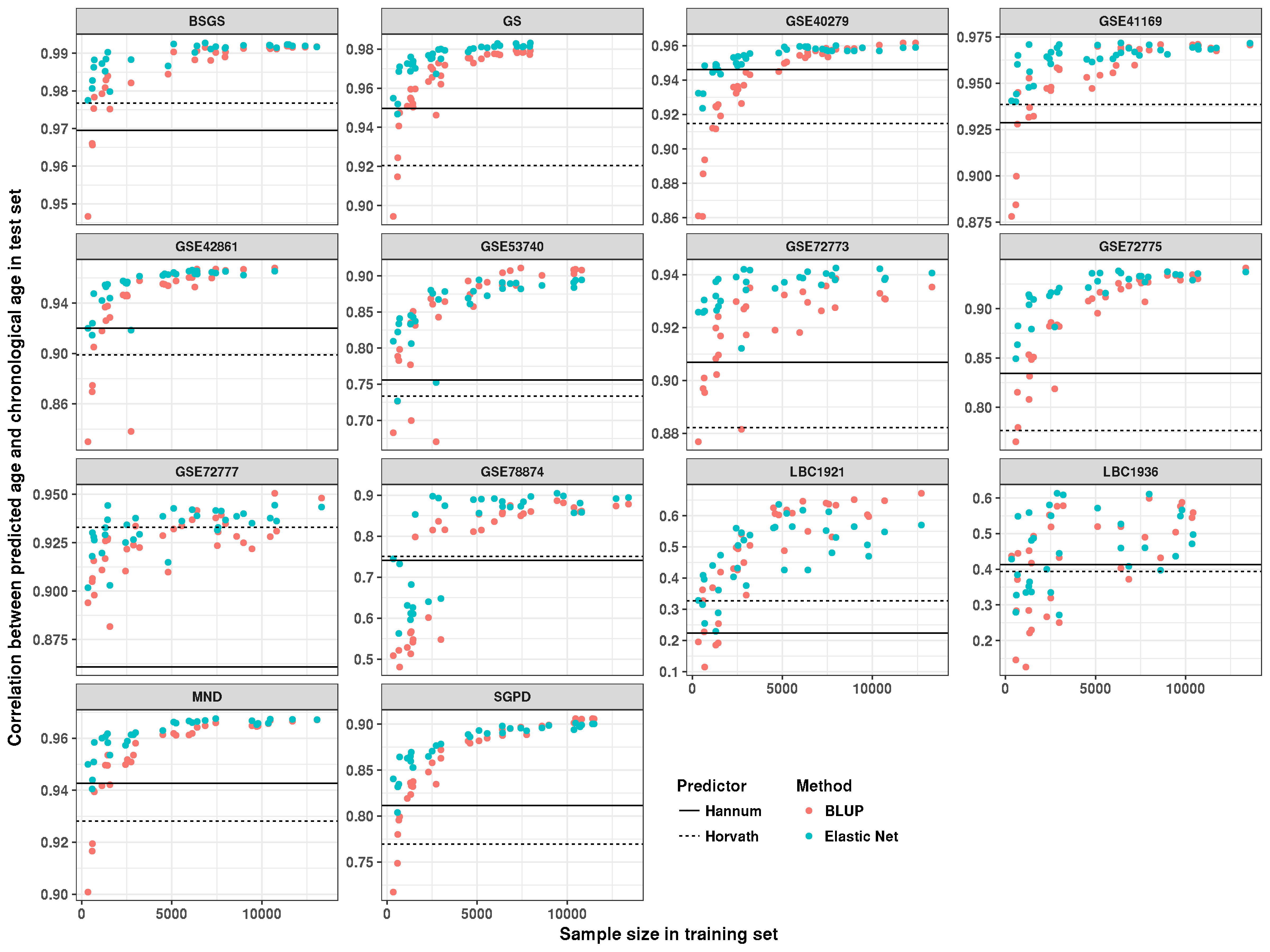


# Figure S3: The comparison between chronological age and predicted age based on different studies. We selected 614 samples from BSGS as the test set. Samples from 13 cohorts (without BSGS) were used as the training dataset. Root mean square error (RMSE) and correlation between predicted age and chronological age were calculated as the prediction accuracy. The solid black line is y = x.


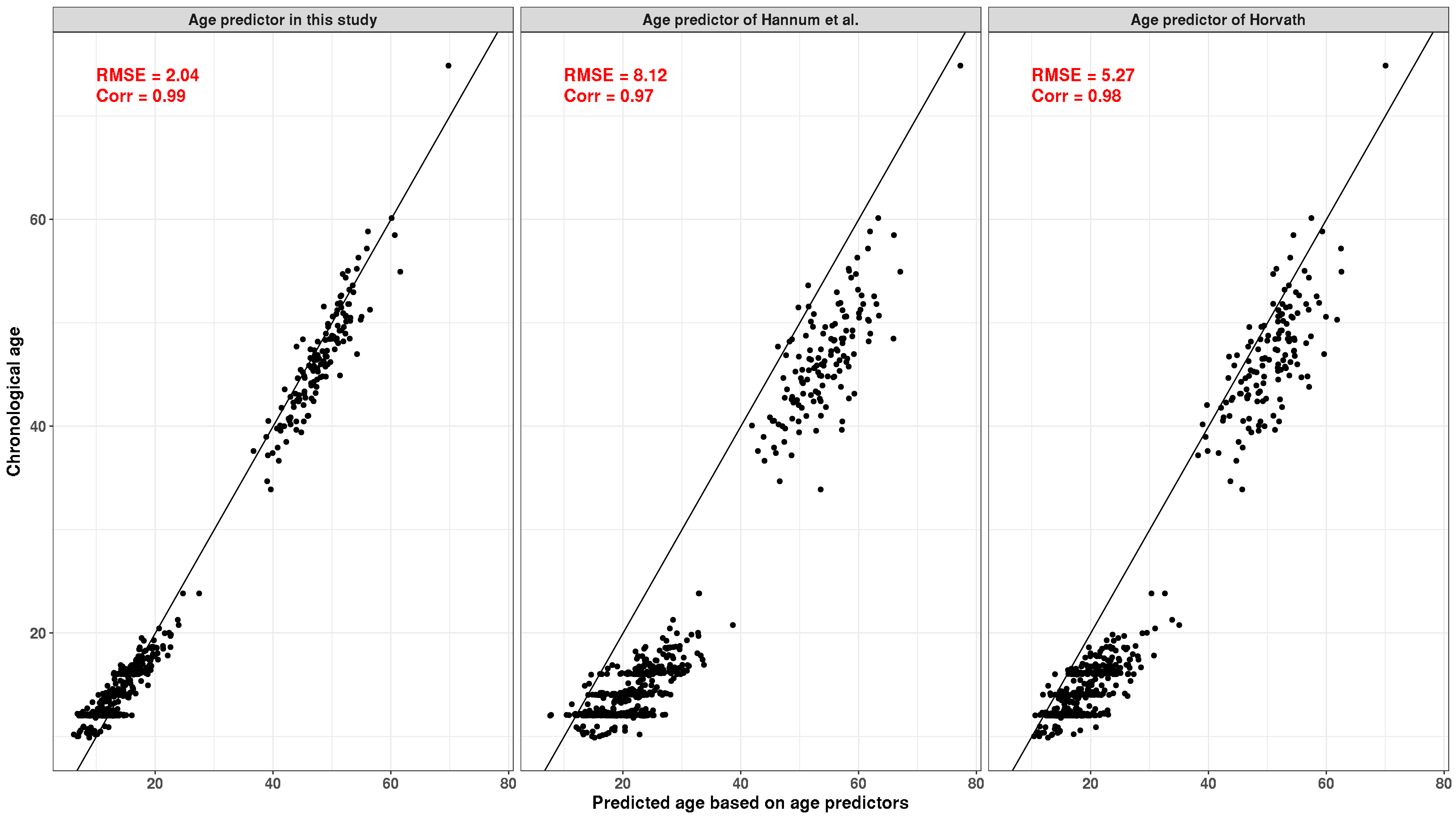


# Figure S4: The difference between BLUP and Elastic Net with the increase of sample size in training data set.


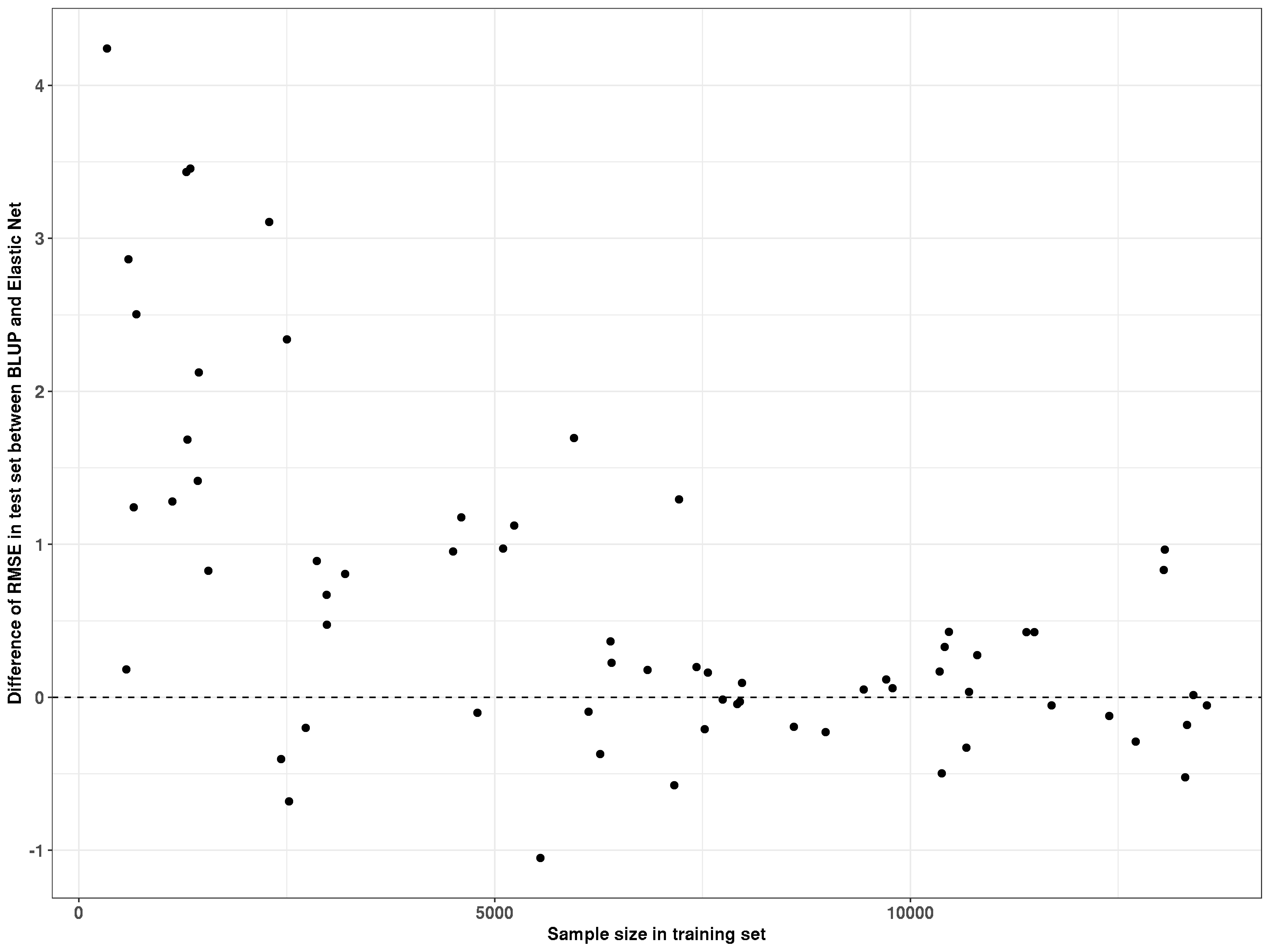


# Figure S5: Improvement of age prediction based on power transformed DNA methylation. We found that the prediction correlation was increased by 3.5% in LBC1921, 8.2% in LBC1936 and 1.3% in SGPD based on power transformed data compared to the result based on raw data. However, the increase was substantially lower in other cohorts (smaller than 1%). Additionally, the smallest correlation was obtained with differing power transformation parameters in each cohort (from 0.2 to 0.7), so that there is not a single power transformation to improve age prediction in all cohorts.


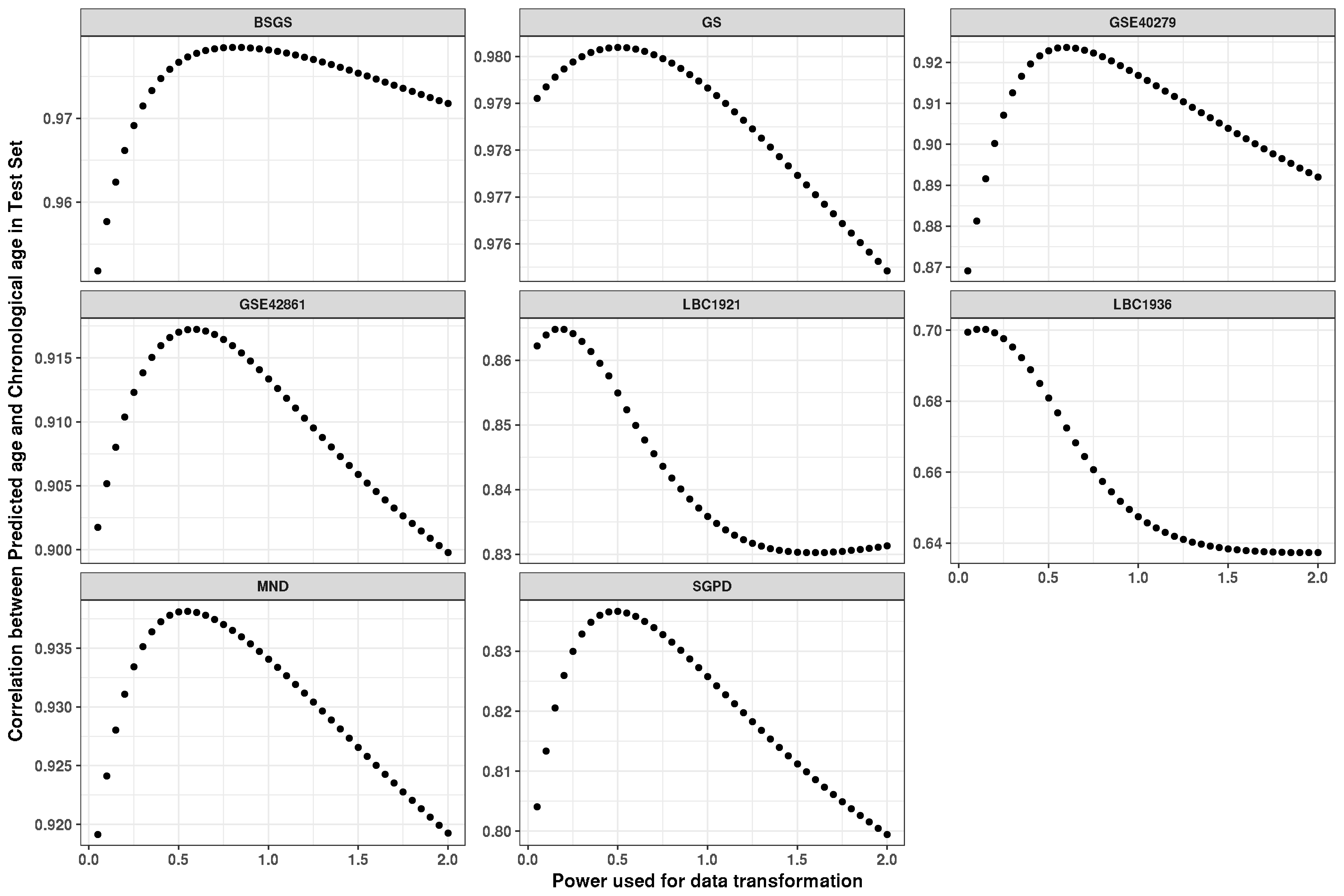


# Figure S6: Comparison of age prediction based on arcsine square root transformed DNA methylation, log transformation, DNA methylation M value, and DNA methylation beta value. 95% confidence interval was added. No improvement was observed using the M values of DNA methylation or using the arcsine square root transformation.


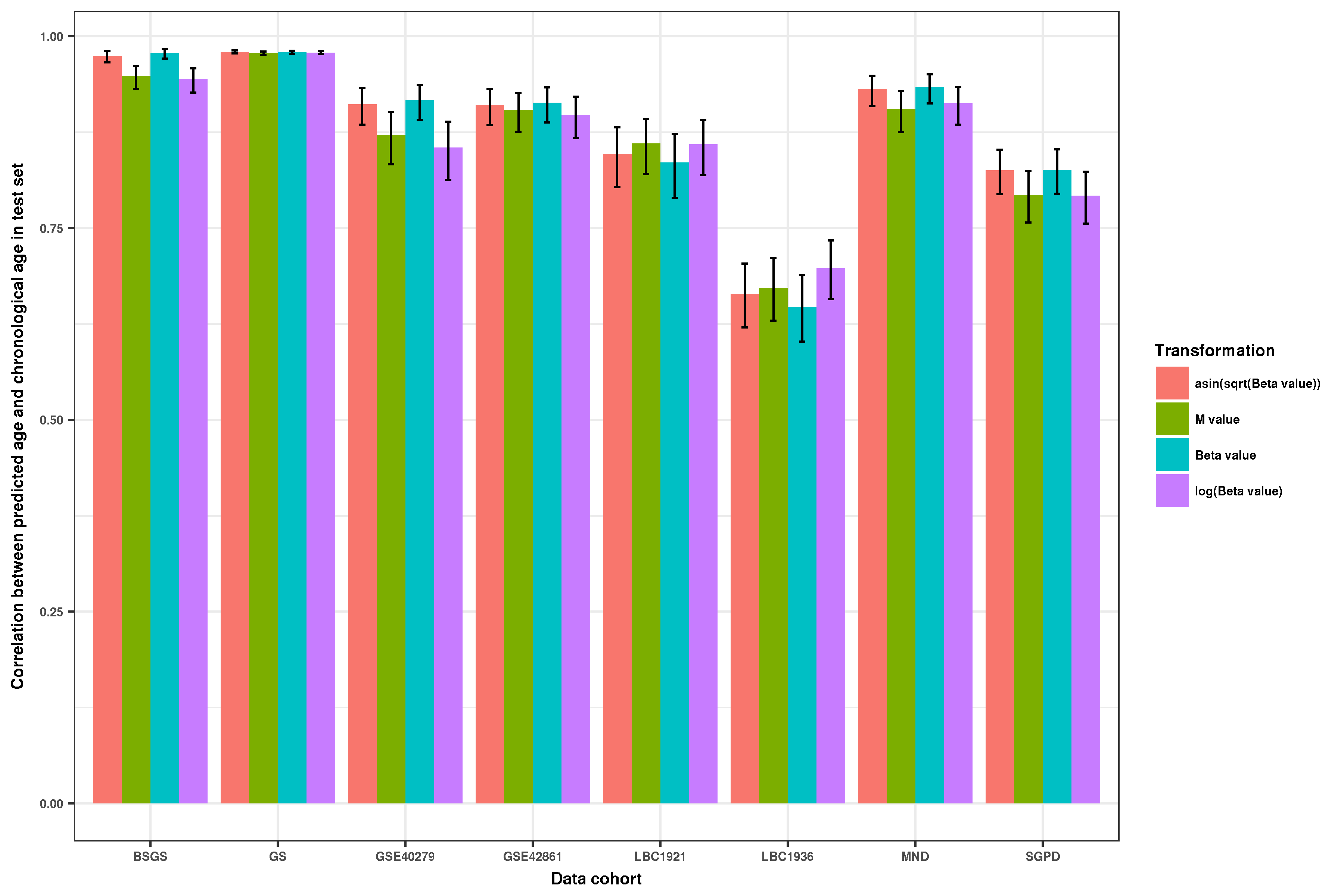


# Figure S7: The comparison between the full probe and pruned probe sets. Two methods including BLUP and Elastic Net were used. Each point represents the difference of prediction accuracy (RMSE or correlation) of each cohort between different probe sets.


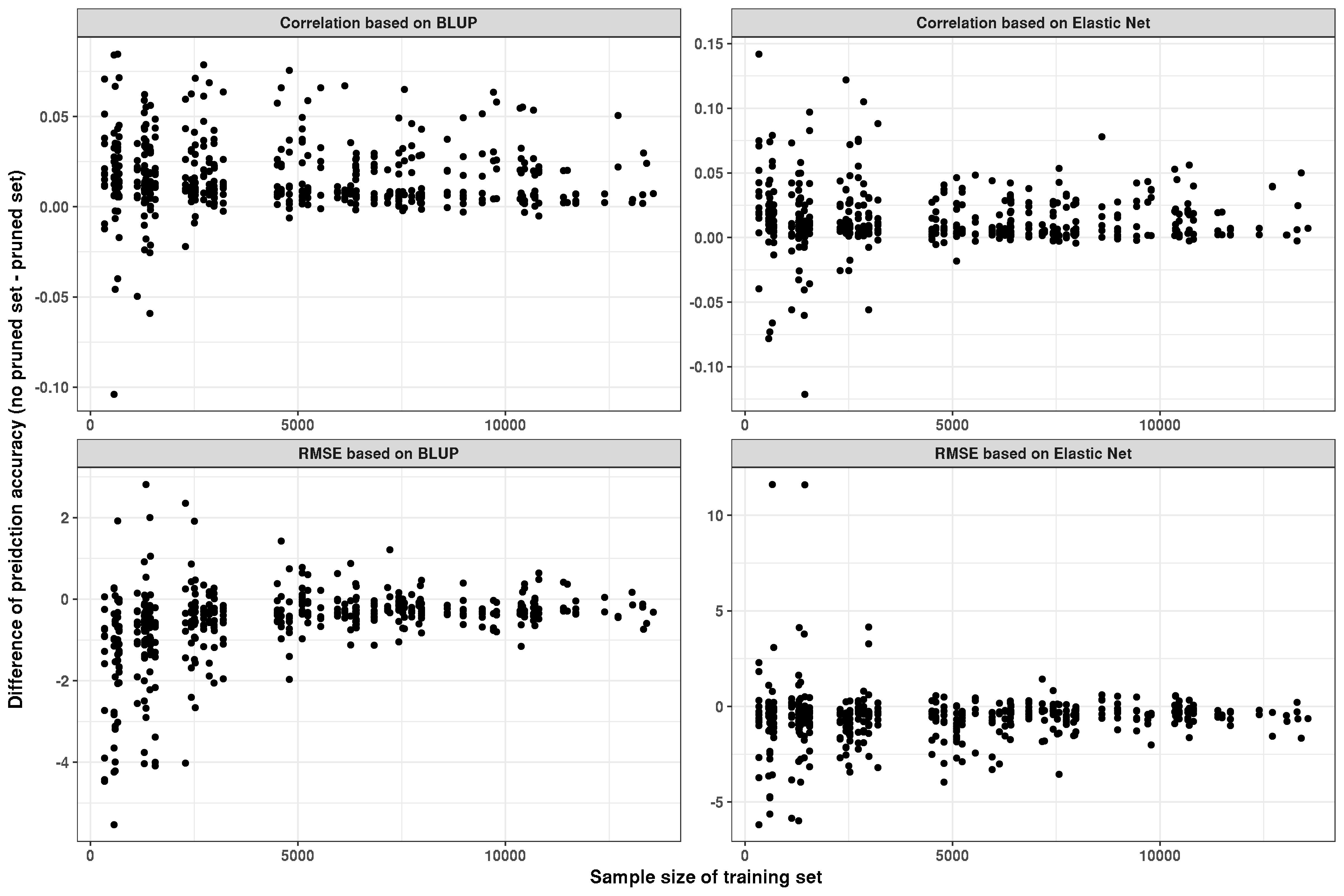


# Figure S8: We compared the probes selected by Elastic Net (based on 13,566 training samples) with those in Horvath’s and Hannum’s age predictors. 11 out of the 514 probes in our analysis were identified in Horvath’s age predictor and 30 in Hannum’s age predictor. In addition, we estimated the squared correlation (R^2^) of DNA methylation between probes selected by Elastic Net and probes from the age predictor of Hannum/Horvath. Apart from pairs with same CpG sites, we found 11 (Elastic Net-Hannum) and 10 (Elastic Net-Horvath) pairs with an R^2^ larger than 0.5, indicating that most of the probes selected by Elastic Net are not strongly correlated with those in the other two predictors. This figure shows the correlation (R^2^) of DNA methylation between probes selected by Elastic Net and probes from age predictors of Hannum and Horvath.


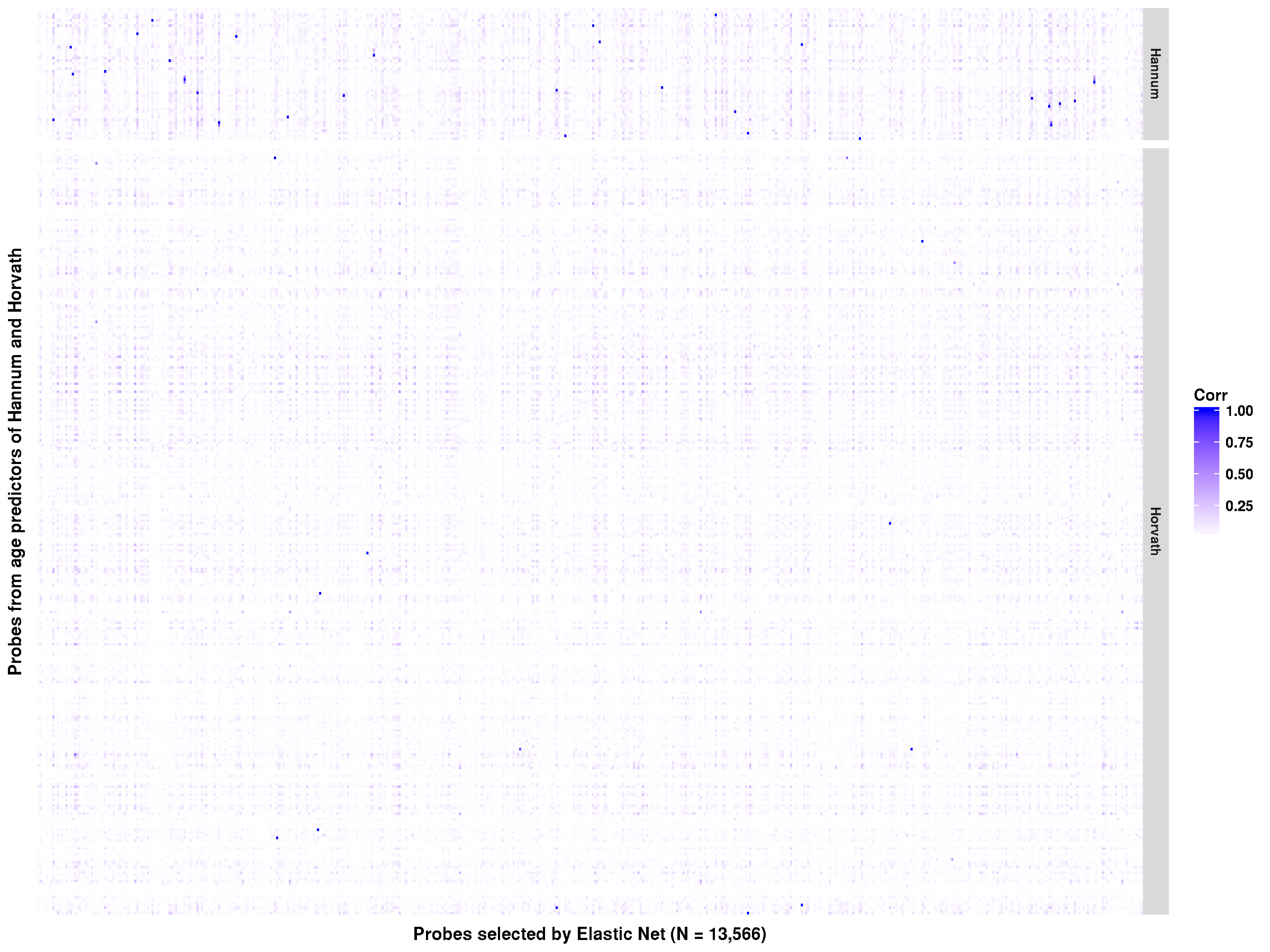


# Figure S9: To quantify whether the probes in the Hannum and Horvarth predictors were necessary for age prediction, we re-built our age predictors by excluding these probes (termed No_HO_HA probe set). No difference in prediction accuracy was found before and after removing these probes for the BLUP based method. Most of the results were similar for the Elastic Net based age predictors.


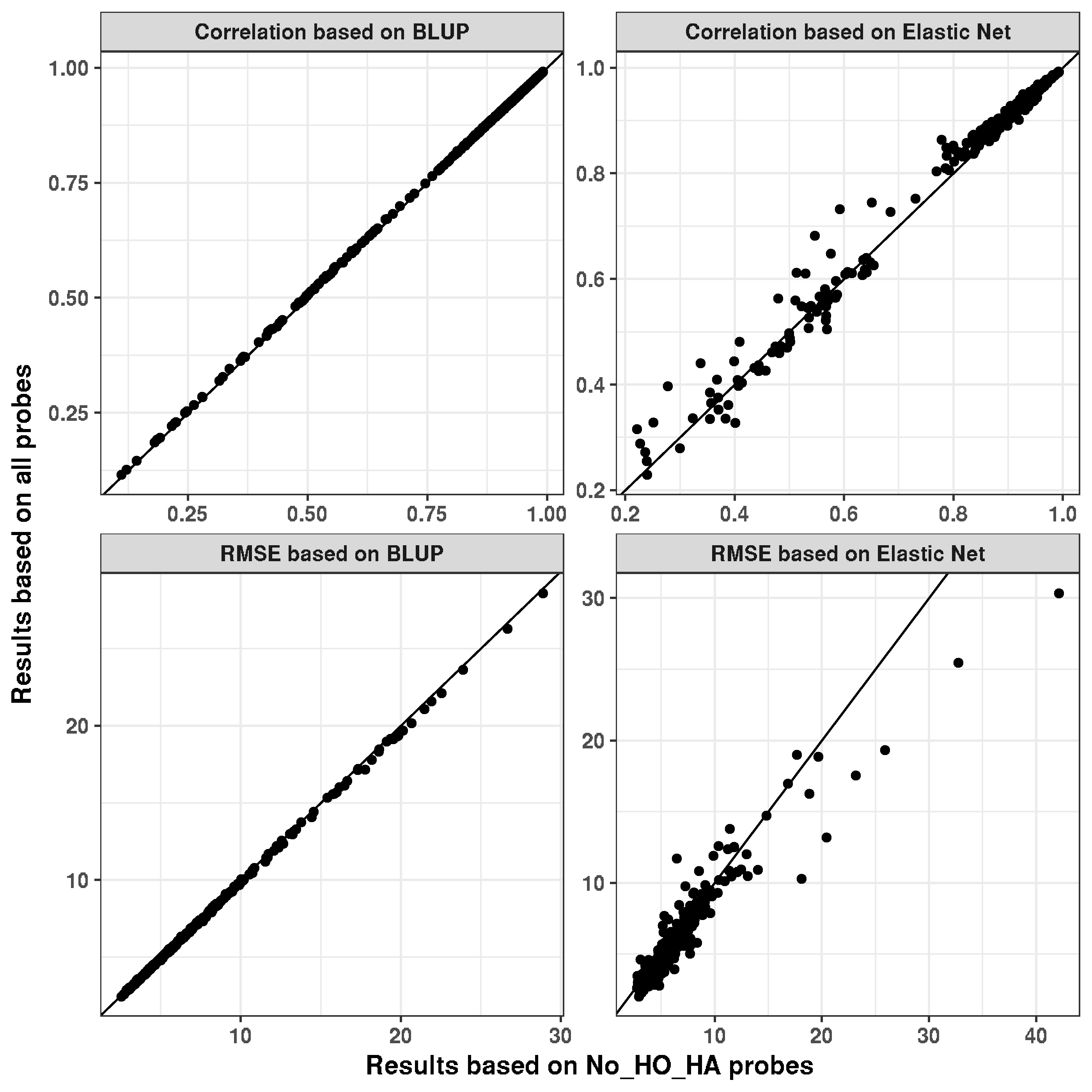


# Figure S10: The prediction accuracy of predictors without DNA methylation probes in Hannum's and Horvath's Age predictors. Root mean square error was calculated for each test set.


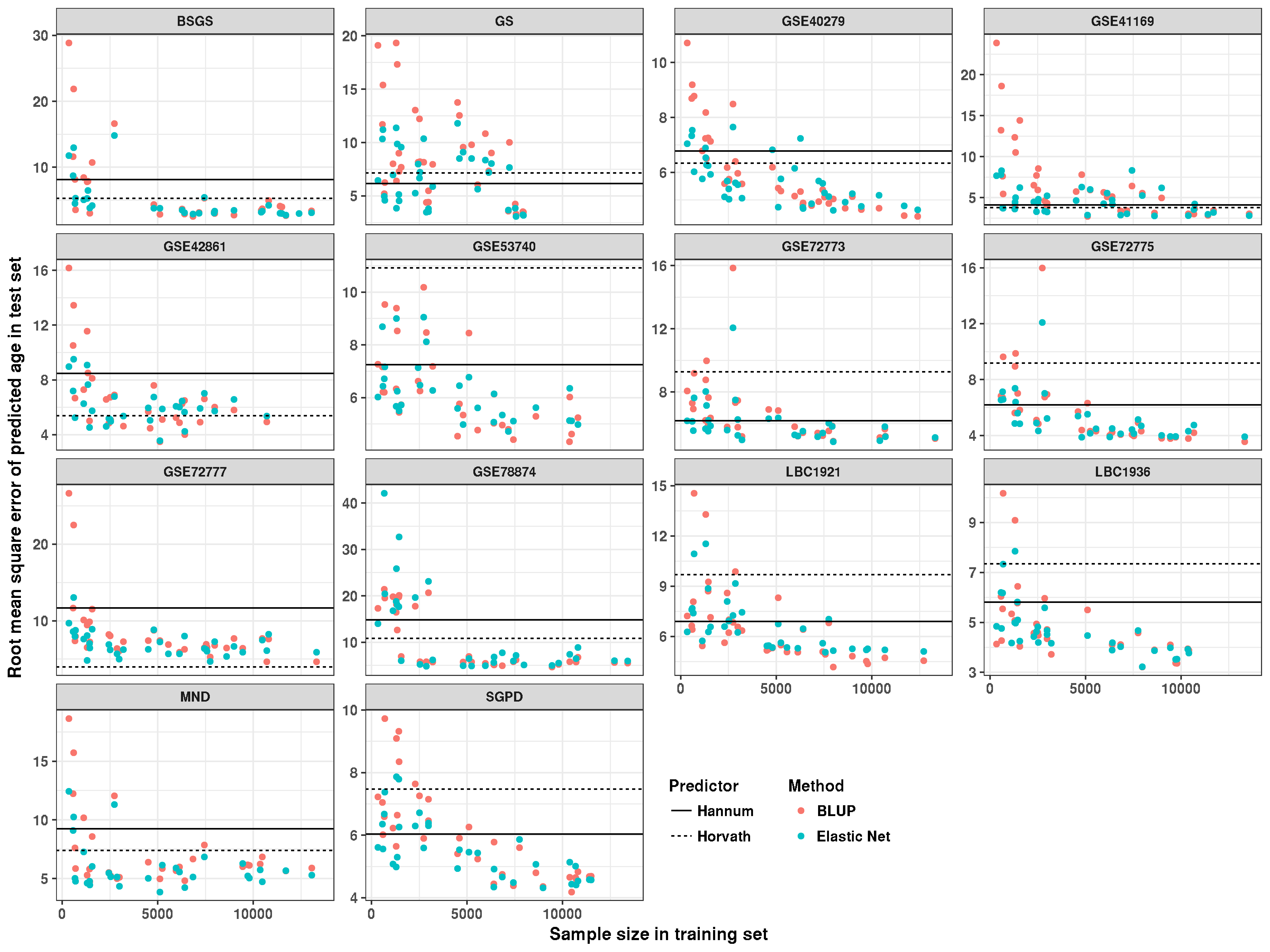


# Figure S11: Relationship between the training sample size and the change of test statistics before and after correcting for the cell counts.


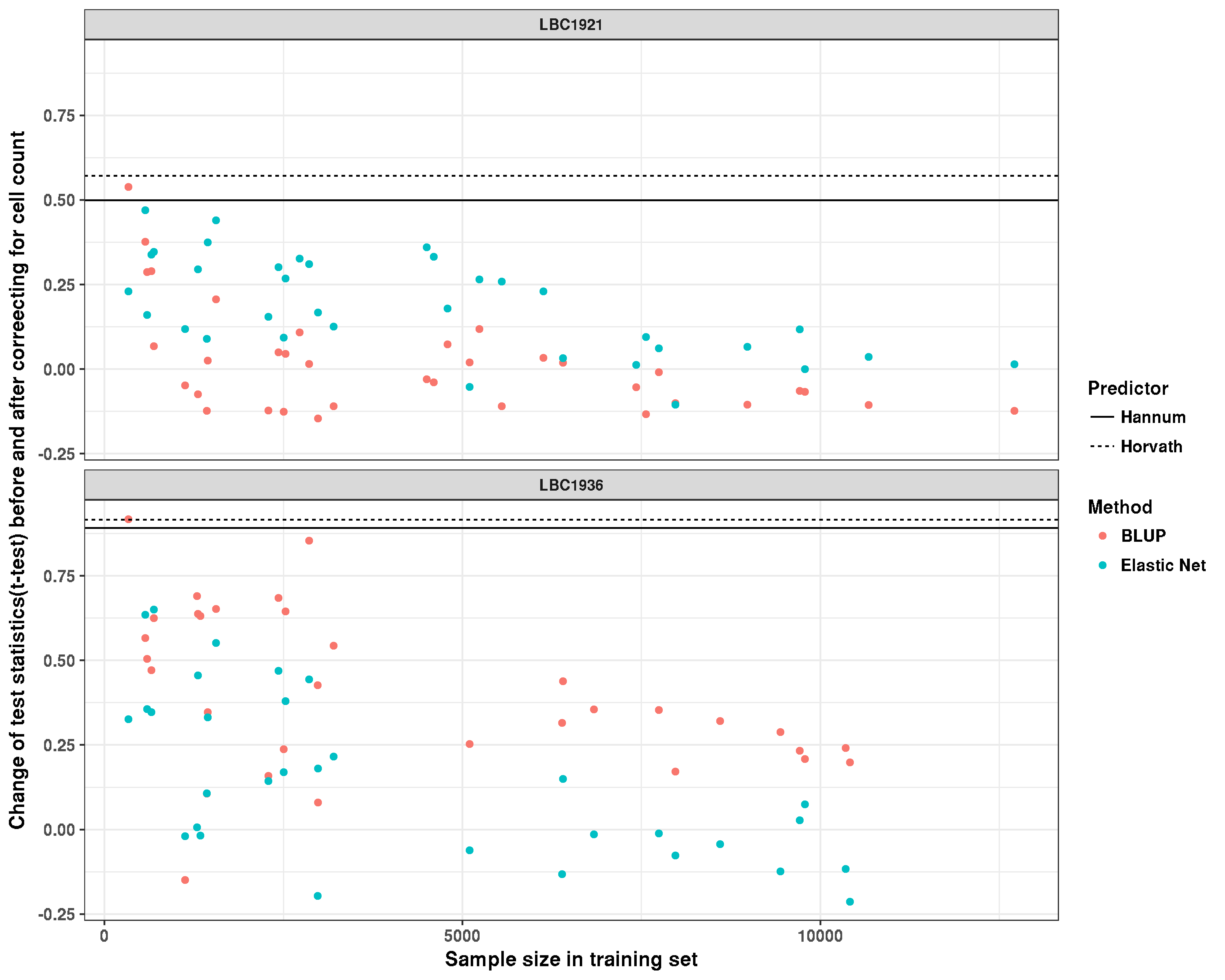


# Table S1: Description of 13 DNA methylation cohorts with non-blood samples

| Cohort^1^ | Sample Size | Number of Samples with valid age | Mean Age(SD) | Age Range | Source |
| --- | --- | --- | --- | --- | --- |
| GSE61431 | 87 | 87 | 61.8 (17.5) | [25, 96] | brain |
| GSE59685 | 451 | 451 | 84.4 (9.3) | [40, 105] | brain |
| GSE80970 | 286 | 286 | 85.8 (8.0) | [70, 108] | brain |
| GSE101961 | 121 | 121 | 38.2 (12.2) | [17, 76] | breast |
| GSE108213 | 85 | 85 | 60.0 (8.9) | [42, 85] | breast |
| GSE61259 | 26 | 26 | 44 (9.1) | [31, 64] | breast |
| GSE88883 | 100 | 100 | 37.2 (13.7) | [18, 82] | breast |
| GSE48325 | 85 | 85 | 47.4 (12.6) | [23, 83] | liver |
| GSE61258 | 79 | 79 | 53.5 (15.5) | [21, 86] | liver |
| GSE61257 | 32 | 32 | 49.7 (13.8) | [31, 79] | adipose |
| GSE90060 | 34 | 34 | 30.1 (3.3) | [23, 36] | endometrium |
| GSE92767 | 54 | 54 | 43.9 (15.1) | [18, 73] | saliva |
| GSE99029 | 57 | 57 | 60.0 (18.0) | [21, 91] | saliva |

^1^ All the cohorts are from the GEO database. GSE61431 (brain)[1], GSE59685 (brain)[2], GSE80970 (brain), GSE101961 (breast)[3], GSE108213 (breast), GSE48325 (liver)[4], GSE61257 (adipose)[5], GSE61258 (liver)[5], GSE61259 (breast)[5], GSE88883 (breast)[6], GSE90060 (endometrium)[7], GSE92767 (saliva)[8], GSE99029 (saliva)[9].

# Table S2: The contributions of three factors (training sample size, the absolute mean age difference between training data set and test data set, and standard deviation of age from training data set) on the prediction accuracy. We fitted a linear regression with RMSE in the test set as the dependent variable, and these three factors as independent variables.

|  |  | **BLUP** | | | **Elastic Net** | | |
| --- | --- | --- | --- | --- | --- | --- | --- |
|  |  | Estimate | SE | P value | Estimate | SE | P value |
| **BSGS** | Sample Size | -4.60E-04 | 2.08E-04 | **3.63E-02**^2^ | -2.91E-04 | 1.19E-04 | **2.23E-02** |
|  | Age Diff^1^ | 4.21E-01 | 1.49E-01 | **8.94E-03** | 2.23E-01 | 8.53E-02 | **1.51E-02** |
|  | SD of age | -4.55E-01 | 2.93E-01 | 1.33E-01 | -1.59E-01 | 1.68E-01 | 3.54E-01 |
| **GSE78874** | Sample Size | -1.15E-03 | 2.08E-04 | **6.84E-06** | -1.07E-03 | 2.68E-04 | **4.16E-04** |
|  | Age Diff | 7.84E-02 | 1.26E-01 | 5.38E-01 | 1.08E-01 | 1.62E-01 | 5.12E-01 |
|  | SD of age | 6.47E-02 | 2.23E-01 | 7.74E-01 | 1.29E-01 | 2.87E-01 | 6.58E-01 |
| **GSE72775** | Sample Size | -4.41E-04 | 1.08E-04 | **3.67E-04** | -2.19E-04 | 6.25E-05 | **1.60E-03** |
|  | Age Diff | 2.04E-02 | 5.67E-02 | 7.22E-01 | 1.38E-02 | 3.27E-02 | 6.76E-01 |
|  | SD of age | -1.25E-01 | 1.05E-01 | 2.44E-01 | -8.59E-02 | 6.06E-02 | 1.67E-01 |
| **MND** | Sample Size | -1.63E-04 | 1.06E-04 | 1.35E-01 | -5.53E-05 | 6.60E-05 | 4.10E-01 |
|  | Age Diff | 2.05E-01 | 7.16E-02 | **8.20E-03** | 1.41E-01 | 4.47E-02 | **3.98E-03** |
|  | SD of age | -2.92E-01 | 1.22E-01 | **2.38E-02** | -1.16E-01 | 7.58E-02 | 1.38E-01 |
| **GSE41169** | Sample Size | -6.72E-04 | 1.22E-04 | **4.66E-06** | -9.74E-05 | 5.11E-05 | 6.57E-02 |
|  | Age Diff | 2.64E-01 | 7.06E-02 | **7.16E-04** | 6.12E-02 | 2.96E-02 | **4.68E-02** |
|  | SD of age | -2.37E-01 | 1.27E-01 | 7.14E-02 | -7.97E-02 | 5.33E-02 | 1.44E-01 |
| **GSE40279** | Sample Size | -3.59E-04 | 3.50E-05 | **9.10E-12** | -1.66E-04 | 2.92E-05 | **2.36E-06** |
|  | Age Diff | 1.49E-02 | 2.09E-02 | 4.80E-01 | -3.95E-02 | 1.74E-02 | **2.98E-02** |
|  | SD of age | -1.45E-01 | 2.88E-02 | **1.56E-05** | -8.30E-02 | 2.39E-02 | **1.48E-03** |
| **GS** | Sample Size | -1.12E-03 | 2.41E-04 | **6.15E-05** | -6.80E-04 | 1.81E-04 | **7.08E-04** |
|  | Age Diff | 5.67E-01 | 1.41E-01 | **3.39E-04** | 4.85E-01 | 1.06E-01 | **6.92E-05** |
|  | SD of age | 5.84E-01 | 1.85E-01 | **3.57E-03** | 4.28E-01 | 1.39E-01 | **4.28E-03** |
| **SGPD** | Sample Size | -3.26E-04 | 3.30E-05 | **6.13E-11** | -1.54E-04 | 2.01E-05 | **1.50E-08** |
|  | Age Diff | 9.52E-02 | 2.07E-02 | **7.01E-05** | 6.23E-02 | 1.26E-02 | **2.67E-05** |
|  | SD of age | -1.16E-02 | 3.46E-02 | 7.39E-01 | 1.63E-02 | 2.10E-02 | 4.46E-01 |
| **GSE72777** | Sample Size | -4.84E-04 | 1.52E-04 | **3.30E-03** | -6.22E-05 | 3.49E-05 | 8.50E-02 |
|  | Age Diff | 1.79E-01 | 8.93E-02 | 5.40E-02 | 4.29E-02 | 2.06E-02 | **4.57E-02** |
|  | SD of age | -3.16E-01 | 1.61E-01 | 5.97E-02 | -1.81E-01 | 3.72E-02 | **3.32E-05** |
| **GSE72773** | Sample Size | -2.84E-04 | 9.58E-05 | **6.48E-03** | -1.13E-04 | 5.64E-05 | 5.60E-02 |
|  | Age Diff | 1.18E-02 | 5.54E-02 | 8.33E-01 | 3.48E-02 | 3.27E-02 | 2.97E-01 |
|  | SD of age | -1.22E-01 | 8.33E-02 | 1.54E-01 | -7.77E-02 | 4.91E-02 | 1.26E-01 |
| **LBC1921** | Sample Size | -4.89E-04 | 6.74E-05 | **3.67E-08** | -2.52E-04 | 4.03E-05 | **6.02E-07** |
|  | Age Diff | 2.26E-01 | 3.62E-02 | **6.37E-07** | 1.41E-01 | 2.16E-02 | **2.74E-07** |
|  | SD of age | -2.36E-01 | 6.45E-02 | **9.29E-04** | -8.60E-02 | 3.85E-02 | **3.31E-02** |
| **GSE42861** | Sample Size | -4.65E-04 | 1.51E-04 | **4.88E-03** | -4.80E-05 | 8.63E-05 | 5.83E-01 |
|  | Age Diff | 9.86E-02 | 9.20E-02 | 2.94E-01 | 1.47E-01 | 5.25E-02 | **9.68E-03** |
|  | SD of age | -1.98E-01 | 1.08E-01 | 7.77E-02 | -1.05E-02 | 6.16E-02 | 8.66E-01 |
| **GSE53740** | Sample Size | -3.15E-04 | 7.05E-05 | **1.35E-04** | -1.73E-04 | 5.01E-05 | **1.89E-03** |
|  | Age Diff | 9.18E-02 | 3.59E-02 | **1.69E-02** | 3.29E-02 | 2.56E-02 | 2.09E-01 |
|  | SD of age | -5.49E-02 | 6.12E-02 | 3.78E-01 | 4.47E-02 | 4.35E-02 | 3.14E-01 |
| **LBC1936** | Sample Size | -3.41E-04 | 5.43E-05 | **8.51E-07** | -2.16E-04 | 2.86E-05 | **3.09E-08** |
|  | Age Diff | 1.42E-01 | 2.89E-02 | **3.39E-05** | 6.45E-02 | 1.52E-02 | **2.22E-04** |
|  | SD of age | -1.40E-01 | 4.51E-02 | **4.28E-03** | -6.86E-02 | 2.38E-02 | **7.40E-03** |

^1^ Age Diff: Absolute Mean Age difference between training data set and test data set.

^2^ P-values samller than 0.05 were marked as bold.

**Table S3:** The contributions of three factors (training sample size, the absolute mean age difference between training data set and test data set, and standard deviation of age from training data set) on the prediction accuracy. We fitted a linear regression with correlation (between predicted age and chronological age) in the test set as the dependent variable and these three factors as independent variables.

|  |  | **BLUP** | | | **Elastic Net** | | |
| --- | --- | --- | --- | --- | --- | --- | --- |
|  |  | Estimate | SE | P value | Estimate | SE | P value |
| **BSGS** | Sample Size | 1.32E-06 | 2.92E-07 | **1.23E-04** | 5.01E-07 | 1.07E-07 | **8.79E-05** |
|  | Age Diff | -4.60E-04 | 2.09E-04 | **3.69E-02** | -2.62E-04 | 7.67E-05 | **2.14E-03** |
|  | SD of age | 8.27E-04 | 4.12E-04 | 5.55E-02 | 2.86E-04 | 1.51E-04 | 7.05E-02 |
| **GSE78874** | Sample Size | 3.11E-05 | 4.48E-06 | **1.54E-07** | 2.13E-05 | 4.02E-06 | **1.21E-05** |
|  | Age Diff | -6.33E-04 | 2.71E-03 | 8.17E-01 | -7.46E-04 | 2.43E-03 | 7.62E-01 |
|  | SD of age | -1.92E-03 | 4.81E-03 | 6.93E-01 | -5.14E-03 | 4.31E-03 | 2.43E-01 |
| **GSE72775** | Sample Size | 1.20E-05 | 1.31E-06 | **8.60E-10** | 4.74E-06 | 8.06E-07 | **2.95E-06** |
|  | Age Diff | -8.33E-04 | 6.86E-04 | 2.35E-01 | -2.52E-05 | 4.21E-04 | 9.53E-01 |
|  | SD of age | 3.21E-03 | 1.27E-03 | **1.79E-02** | 1.61E-03 | 7.82E-04 | **4.94E-02** |
| **MND** | Sample Size | 2.67E-06 | 4.62E-07 | **4.42E-06** | 1.16E-06 | 2.14E-07 | **1.12E-05** |
|  | Age Diff | -4.59E-04 | 3.13E-04 | 1.54E-01 | -2.17E-04 | 1.45E-04 | 1.47E-01 |
|  | SD of age | 1.08E-03 | 5.31E-04 | 5.19E-02 | 3.57E-04 | 2.46E-04 | 1.58E-01 |
| **GSE41169** | Sample Size | 4.08E-06 | 5.31E-07 | **9.31E-09** | 1.21E-06 | 2.04E-07 | **1.43E-06** |
|  | Age Diff | -1.18E-03 | 3.07E-04 | **5.39E-04** | -6.32E-04 | 1.18E-04 | **7.08E-06** |
|  | SD of age | 1.10E-03 | 5.54E-04 | 5.47E-02 | 3.26E-04 | 2.13E-04 | 1.35E-01 |
| **GSE40279** | Sample Size | 6.25E-06 | 6.58E-07 | **5.73E-11** | 1.83E-06 | 2.62E-07 | **5.60E-08** |
|  | Age Diff | 2.41E-05 | 3.92E-04 | 9.51E-01 | 1.27E-04 | 1.56E-04 | 4.23E-01 |
|  | SD of age | 2.15E-03 | 5.40E-04 | **3.59E-04** | 5.52E-04 | 2.15E-04 | **1.49E-02** |
| **GS** | Sample Size | 6.27E-06 | 7.52E-07 | **2.02E-09** | 2.75E-06 | 3.47E-07 | **6.07E-09** |
|  | Age Diff | -7.37E-04 | 4.39E-04 | 1.03E-01 | -4.74E-04 | 2.02E-04 | 2.57E-02 |
|  | SD of age | 9.45E-04 | 5.77E-04 | 1.11E-01 | 2.75E-04 | 2.66E-04 | 3.10E-01 |
| **SGPD** | Sample Size | 1.01E-05 | 9.47E-07 | **9.83E-12** | 4.97E-06 | 5.79E-07 | **1.43E-09** |
|  | Age Diff | 7.05E-04 | 5.92E-04 | 2.43E-01 | 4.28E-04 | 3.62E-04 | 2.46E-01 |
|  | SD of age | 3.73E-03 | 9.91E-04 | **7.26E-04** | 9.82E-04 | 6.06E-04 | 1.15E-01 |
| **GSE72777** | Sample Size | 2.48E-06 | 4.27E-07 | **2.35E-06** | 1.40E-06 | 3.51E-07 | **3.94E-04** |
|  | Age Diff | -3.91E-04 | 2.51E-04 | 1.30E-01 | -4.73E-04 | 2.07E-04 | **2.96E-02** |
|  | SD of age | 1.21E-03 | 4.54E-04 | **1.23E-02** | 6.08E-04 | 3.73E-04 | 1.14E-01 |
| **GSE72773** | Sample Size | 2.97E-06 | 5.44E-07 | **9.82E-06** | 1.05E-06 | 2.47E-07 | **2.44E-04** |
|  | Age Diff | 3.22E-04 | 3.15E-04 | 3.15E-01 | 1.10E-04 | 1.43E-04 | 4.48E-01 |
|  | SD of age | 1.42E-03 | 4.73E-04 | **5.88E-03** | 6.87E-04 | 2.15E-04 | **3.62E-03** |
| **LBC1921** | Sample Size | 4.22E-05 | 4.03E-06 | **1.03E-11** | 2.27E-05 | 3.12E-06 | **3.59E-08** |
|  | Age Diff | -1.02E-02 | 2.16E-03 | **4.72E-05** | -1.06E-02 | 1.68E-03 | **4.83E-07** |
|  | SD of age | 1.26E-02 | 3.85E-03 | **2.63E-03** | 1.19E-02 | 2.99E-03 | **3.93E-04** |
| **GSE42861** | Sample Size | 7.92E-06 | 1.59E-06 | **3.54E-05** | 3.45E-06 | 6.40E-07 | **1.19E-05** |
|  | Age Diff | -1.27E-03 | 9.68E-04 | 2.02E-01 | -1.76E-04 | 3.90E-04 | 6.55E-01 |
|  | SD of age | 2.95E-03 | 1.14E-03 | **1.52E-02** | 1.53E-03 | 4.57E-04 | **2.54E-03** |
| **GSE53740** | Sample Size | 1.36E-05 | 2.55E-06 | **1.36E-05** | 7.09E-06 | 1.60E-06 | **1.52E-04** |
|  | Age Diff | 2.81E-03 | 1.30E-03 | **4.01E-02** | 1.54E-03 | 8.16E-04 | 7.00E-02 |
|  | SD of age | 1.82E-03 | 2.22E-03 | 4.19E-01 | 1.69E-03 | 1.39E-03 | 2.35E-01 |
| **LBC1936** | Sample Size | 1.93E-05 | 6.39E-06 | **5.25E-03** | 4.84E-06 | 5.22E-06 | 3.62E-01 |
|  | Age Diff | 4.93E-03 | 3.40E-03 | 1.58E-01 | 6.56E-03 | 2.78E-03 | **2.55E-02** |
|  | SD of age | -6.86E-03 | 5.31E-03 | 2.07E-01 | -6.58E-03 | 4.34E-03 | 1.40E-01 |

# Reference

1. Pidsley R, Viana J, Hannon E, Spiers H, Troakes C, Al-Saraj S, Mechawar N, Turecki G, Schalkwyk LC, Bray NJ, Mill J: **Methylomic profiling of human brain tissue supports a neurodevelopmental origin for schizophrenia.** *Genome Biol* 2014, **15:**483. GEO: GSE61431

2. Lunnon K, Smith R, Hannon E, De Jager PL, Srivastava G, Volta M, Troakes C, Al-Sarraj S, Burrage J, Macdonald R, et al: **Methylomic profiling implicates cortical deregulation of ANK1 in Alzheimer's disease.** *Nat Neurosci* 2014, **17:**1164-1170. GEO: GSE59685

3. Song MA, Brasky TM, Weng DY, McElroy JP, Marian C, Higgins MJ, Ambrosone C, Spear SL, Llanos AA, Kallakury BVS, et al: **Landscape of genome-wide age-related DNA methylation in breast tissue.** *Oncotarget* 2017, **8:**114648-114662. GEO: GSE101961

4. Ahrens M, Ammerpohl O, von Schonfels W, Kolarova J, Bens S, Itzel T, Teufel A, Herrmann A, Brosch M, Hinrichsen H, et al: **DNA methylation analysis in nonalcoholic fatty liver disease suggests distinct disease-specific and remodeling signatures after bariatric surgery.** *Cell Metab* 2013, **18:**296-302. GEO: GSE48325

5. Horvath S, Erhart W, Brosch M, Ammerpohl O, von Schonfels W, Ahrens M, Heits N, Bell JT, Tsai PC, Spector TD, et al: **Obesity accelerates epigenetic aging of human liver.** *Proc Natl Acad Sci U S A* 2014, **111:**15538-15543. GEO: GSE61257, GSE61258 and GSE61259

6. Johnson KC, Houseman EA, King JE, Christensen BC: **Normal breast tissue DNA methylation differences at regulatory elements are associated with the cancer risk factor age.** *Breast Cancer Research* 2017, **19**. GEO: GSE88883

7. Kukushkina V, Modhukur V, Suhorutsenko M, Peters M, Magi R, Rahmioglu N, Velthut-Meikas A, Altmae S, Esteban FJ, Vilo J, et al: **DNA methylation changes in endometrium and correlation with gene expression during the transition from pre-receptive to receptive phase.** *Sci Rep* 2017, **7:**3916. GEO: GSE90060

8. Hong SR, Jung SE, Lee EH, Shin KJ, Yang WI, Lee HY: **DNA methylation-based age prediction from saliva: High age predictability by combination of 7 CpG markers.** *Forensic Sci Int Genet* 2017, **29:**118-125. GEO: GSE92767

9. Gopalan S, Carja O, Fagny M, Patin E, Myrick JW, McEwen LM, Mah SM, Kobor MS, Froment A, Feldman MW, et al: **Trends in DNA Methylation with Age Replicate Across Diverse Human Populations.** *Genetics* 2017, **206:**1659-1674. GEO: GSE99029
